# Micronutrient's deficiency in India: a systematic review and meta-analysis

**DOI:** 10.1017/jns.2021.102

**Published:** 2021-12-21

**Authors:** U. Venkatesh, Akash Sharma, Velmurugan A. Ananthan, Padmavathi Subbiah, R. Durga, CSIR Summer Research training team

**Affiliations:** 1 Department of Community Medicine & Family Medicine, All India Institute of Medical Sciences, Gorakhpur, India; 2 Rush University Medical Center, Chicago, IL, USA; 3 Department of Community Medicine, Government Theni Medical College, Theni, Tamil Nadu, India; 4 Model Rural Health Research Unit, ICMR-NIE, Kallur, Tirunelveli, Tamil Nadu, India; 5 Department of Paediatric and Preventive Dentistry, Faculty of Dental Sciences, King George Medical University, Lucknow, India

**Keywords:** Iodine deficiency, Iron deficiency, Meta-analysis, Micronutrient deficiency, Vitamin A deficiency, Vitamin B_12_ deficiency, Vitamin D deficiency, CI, confidence interval, IDD, iodine deficiency disorders, NFHS, National Family Health Survey, VAD, vitamin A deficiency, VDD, vitamin D deficiency, WHO, World Health Organization

## Abstract

India is coming to grips with a stage of nutrition transition. According to the Food Safety and Standards Authority of India (FSSAI), preventable micronutrient deficiency is arising public health precedence in India. However, the foremost public health concern is the lack of national prevalence data. The present study was carried out to estimate the pooled age-wise prevalence of six preventable micronutrient deficiencies (vitamin A, vitamin B_12_, vitamin D, iron, iodine and folic acid) in India. A systematic review was carried out on PubMed and Global Index Medicus databases using the Boolean search strategy. Statistical analyses were done using R software, version 3.6. 2. PRISMA guidelines were strictly adhered to during the review. A preliminary literature search yielded 4302 articles; however, 270 original research articles were found eligible to be included in quantitative synthesis. The estimated overall prevalence was 17 % [95 % confidence interval (CI) 0⋅07, 0⋅26] for iodine deficiency, 37 % (95 % CI 0⋅27, 0⋅46) for folic acid deficiency, 54 % (95 % CI 0⋅49, 0⋅59) for iron deficiency, 53 % (95 % CI 0⋅41, 0⋅64) for vitamin B_12_ deficiency, 19 % (95 % CI 0⋅09, 0⋅29) for vitamin A deficiency and 61 % (95 % CI 0⋅07, 0⋅26) for vitamin D with high heterogeneity. We classified the population into infants (0–5 years), adolescents (<18 years), adults (>18 years) and pregnant women. Iron deficiency was most prevalent (61 %) in pregnant women. The results of the present study reinforce the data on micronutrient deficiency in India and warrant the immediate need for further active public health interventions to address these deficiencies. The study is registered with PROSPERO (CRD42020205043).

## Introduction

The World Health Organization (WHO) defines ‘Micronutrients’ as compounds required in very smaller amounts, <100 mg/d^(1,2)^. Micronutrients include vitamins and minerals. They are vital for the production of hormones, enzymes and other substances that manage growth and development^(1,2)^. Diseases caused by deficiencies of iron (anaemia), iodine [(iodine deficiency (IDD) disorders such as goitre and hypothyroidism] and vitamin A [vitamin A deficiency (VAD) disorders such as blindness] are considered major public health problems. The untoward outcomes of ‘Micronutrient Malnutrition’ or ‘Hidden Hunger’ are earnest and include early death, impoverished health, vision effects, stunted growth, mental malady, learning impairment and lassitude^(3)^. According to the WHO, 45 % of deaths in children aged <5 years are linked to undernutrition^(2)^. Globally, in low-income countries, iodine, iron and vitamin A are considered vital micronutrients for health as their deficiency affects children and pregnant women, and 42 % of children aged <5 years and 40 % of pregnant women are anaemic^(1,2)^. The WHO and UNICEF reckoned it in a worldwide assessment estimated that only 66 % of households had iodised salt access, nearly 190 million children of pre-school age and 19⋅1 million pregnant women are deficient in vitamin A with more than 2 million people are flawed of other key micronutrients^(4,5)^.

India is in a stage of nutrition transition. Being overweight and underweight are two common predicaments, but irrespective of them, micronutrient deficiency is at its peak and the leading reason may be the cereal-based food practices in India^(6–8)^. Nutritional deficiencies contributed to 0⋅5 % [95 % confidence interval (CI) 0⋅4 %, 0⋅6 %] of total deaths in India in 2016^(9)^. In India, the National Family Health Survey-4 (NFHS-4) revealed that India has the highest burden of anaemia worldwide^(10)^. The prevalence of anaemia was 58⋅6 % in children, 53⋅2 % in non-pregnant women and 50⋅4 % in pregnant women in 2016^(10)^. According to the Comprehensive National Nutrition Survey of children (CNNSC) between 0 and 19 years in 2019 in India, zinc deficiency was observed in 19 % of pre-school children and 32 % of adolescents, whereas 23 % of pre-school children and 37 % of adolescents were deficient in folate^(11)^. Vitamin B_12_, vitamin A and vitamin D deficiencies (VDDs) range between 14 and 31 % for pre-school children and adolescents. In a community-based cross-sectional study in rural India from eight states, the prevalence of Bitot's spots was 0⋅8 %, while the prevalence of anaemia was 67 % among pre-school children and 69 % among adolescents, and the prevalence of IDD measured by Goitre prevalence was 3⋅9 %^(12)^.

The government of India has launched several schemes and programmes in the wake of micronutrient deficiency, but the problem still exists in a large segment of the population. Food fortification, dietary diversification, nutritional education, micronutrient supplementation, maintenance of environmental sanitation and hygiene are the various available measures taken to tackle the problem of micronutrient malnutrition. But the results have not been satisfactory, and national nutritional programmes have failed to achieve the goals^(13)^. Although anaemia control programme has been running for around 50 years, NFHS-4 data have revealed that India has the highest burden of anaemia worldwide^(10)^. The focus is now primarily on the fortification of foods with essential micronutrients in India. Food fortification of essential micronutrients like iron, folic acid, vitamin B_12_, iodine, vitamin A and vitamin D is being done in India as per the Food Safety and Standards Authority of India (FSSAI) reports^(14,15)^.

Micronutrient malnutrition is a serious public health problem in India in spite of the efforts undertaken by several national health programmes and schemes. Unfortunately, micronutrient supplementation, food fortification and other strategies have been only partially effective. Hence, it is indispensable to determine the type of deficiency and make age-specific recommendations along with the fortification of the foods for the same. The foremost public health concern is the lack of national prevalence data. Although various surveys are being conducted nationwide like NFHS, Annual Health Survey (AHS), National Nutrition Monitoring Bureau (NNMB) and District Level Household Survey (DLHS) to evaluate and estimate nutritional status in various parts of India, they are not recommendable nationwide because of their diversity and limitations in sampling and methodology. The national nutritional surveys have also mainly focused on anthropometric measurements and dietary intakes rather than on the prevalence of micronutrient deficiencies because of practical constraints. Despite everything, there is a paucity of high-quality evidence regarding preventable micronutrient deficiency in India. Preventable micronutrient deficiency is arising public health precedence in India. Hence, the problem of micronutrient malnutrition needs effective policies and strategies. In order to define the priority areas for future interventions and the evaluation of current strategies, the void of nationally representative data on micronutrient deficiency must be filled. There is a need to integrate readily available nationwide data to reach any denouement and understand the role of fortification of foods to combat micronutrient deficiency for making stronger and age-specific recommendations. The present systematic review and meta-analysis were carried out with the basic objective to find the overall prevalence and age-wise estimates of deficiency of the six major micronutrients in India: iron, folic acid, vitamin B_12_, iodine, vitamin A and vitamin D.

## Methodology

The prime objective of the present study is to determine the prevalence of preventable micronutrient deficiencies like vitamin A, vitamin B_12_, vitamin D, iron, iodine and folic acid in several age groups that the FSSAI is promoting for the fortification of food in India. The current systematic review and meta-analysis study has strictly adhered to the Preferred Reporting Items for Systematic Reviews and Meta-Analyses (PRISMA) checklist^(16)^. The study protocol was registered with the PROSPERO, University of York (CRD42020205043).

### Eligibility criteria

Studies were limited to primary research papers using any methods, articles published in English and only original studies conducted in India between 2015 and 2020 that were relevant to the present study objective. The recent nationally representative data available was NFHS-4 2015–2016. Hence, we considered the papers published since 2015. Studies that provided data either on the deficiency of micronutrients were only included. Studies carried out in various states of India were also included.

Research articles other than the original research articles were excluded. Abstract only papers, preceding papers, editorial, author responses, thesis, books, case reports, case series, irrelevant, unpublished material available and duplicated articles were disqualified.

### Search strategy

The present study was launched on 26 July 2020 by searching and screening articles and abstracts from PubMed and GIM from 2015 to 2020 which carried out surveys to find out micronutrient deficiencies in several parts of India, including varying populations from different areas and backgrounds. The Boolean search strategy was applied across both databases at PubMed and Global Index Medicus. Various search strings or keywords were used to search the database. To search studies related to ‘IDD’, keywords and a string ‘(Iodine) AND (Deficiency OR prevalence OR Determinants) AND (India)’ were used to search the articles. Similarly, for ‘Iron’ keywords and string ‘(Iron OR Anemia) AND (Deficiency OR prevalence OR Determinants) AND (India)’, for ‘Folic acid’ keywords and strings ‘(Folate OR Folic acid OR Vitamin B9) AND (Deficiency OR prevalence OR Determinants) AND (India)’, for ‘Vitamin B_12_’ keyword and string ‘(Vitamin B_12_) AND (Deficiency OR prevalence OR Determinants) AND (India)’, for ‘Vitamin A’ keyword and string ‘(Vitamin A) AND (Deficiency OR prevalence OR Determinants) AND (India)’ and for ‘Vitamin D’ keyword and string ‘(Vitamin D) AND (Deficiency OR prevalence OR Determinants) AND (India)’ were used to search the articles for respective micronutrients.

According to inclusion criteria, the cited articles relevant to the studies were manually searched on several search engines. Only potentially appropriate and full-text articles were reviewed. If the abstract of the article alone was not able to make the decision, then the full text was also reviewed. The research team manually searched the reference list of all selected articles to find out if any admissible studies were ignored during a search. If some data were unavailable in full text, the authors also contacted investigators to acquire additional data. Two groups of experts separately screened and sorted the titles and abstracts, and any disagreements in the selection of study were resolved by mutual consent. If multiple studies were utilised in the same dataset or cohort, then the most comprehensive study with the largest number of participants was selected for the present study.

### Data extraction and quality assessment

Two independent reviewers performed data extraction. All the data collected from searched records were entered in an excel sheet with essential information such as the name of the author and the journal, year of publication, DOI, URL link and the abstract for screening. The following variables were considered for study: author, year of study and publication, place of study, study design, study setting, sample techniques, sample size and age group involved in the study population, micronutrient deficient samples, prevalence and 95 % CI. The National Institutes of Health (NIH) tool for observational and cross-sectional studies was utilised for quality assessment. The risk of bias was evaluated using fourteen items. Each of the fourteen items was rated into dichotomous variables: yes, no or not applicable. Any dilemma regarding the decisions about inclusion of studies, interpretation of data was resolved by discussions and consensus among the investigators. The quality of the study was reported as good, fair and poor. Quality was rated as 0 for poor (zero to four out of fourteen questions), i for fair (five to ten out of fourteen questions) or ii for good (eleven to fourteen out of fourteen questions).

### Statistical analysis

Title and abstract were screened and extracted data in the excel sheet were double-checked by two independent group members. The main outcome measures were the prevalence of preventable micronutrient deficiencies in India. A subgroup analysis for different age groups depending on the micronutrients was also done. For folic acid, iodine, vitamin B_12_ and vitamin D, we divided age groups into adolescents (0–18 years) and adults (>18 years). However, in the case of vitamin A we used <5 years and >5 years groups, and for iron it was children (<5 years), adolescents (between 5 and 18 years), adults (>18 years) and pregnant women. The basis of division is when these micronutrient deficiencies have more health implications and the availability of age-wise distribution of data in research studies. For overlapping groups and when no specific age groups were mentioned in the study, we grouped them together in a non-specific group. A value of I2 0 % indicates no observed heterogeneity, while a value of 100 % indicates significant heterogeneity. For this review, we determined that I2 values >75 % were indicative of significant heterogeneity, warranting analysis with a random effect model as opposed to the fixed effect model to adjust for the observed variability. Statistical analyses were done using R software, version 3.6. 2. A *P*-value of <0⋅05 was considered statistically significant.

Using Egger's regression test, we assessed publication bias where a *P*-value of <0⋅10 is regarded as statistically significant. Begg's funnel plot is drawn to represent publication bias graphically in the case of studies numbering 10 or more than 10. A trim and fill method given by Duvall and Tweedie was applied to add missing studies (Supplementary Fig. S1).

## Results

### Search results and study characteristics

**Fig. 1.**
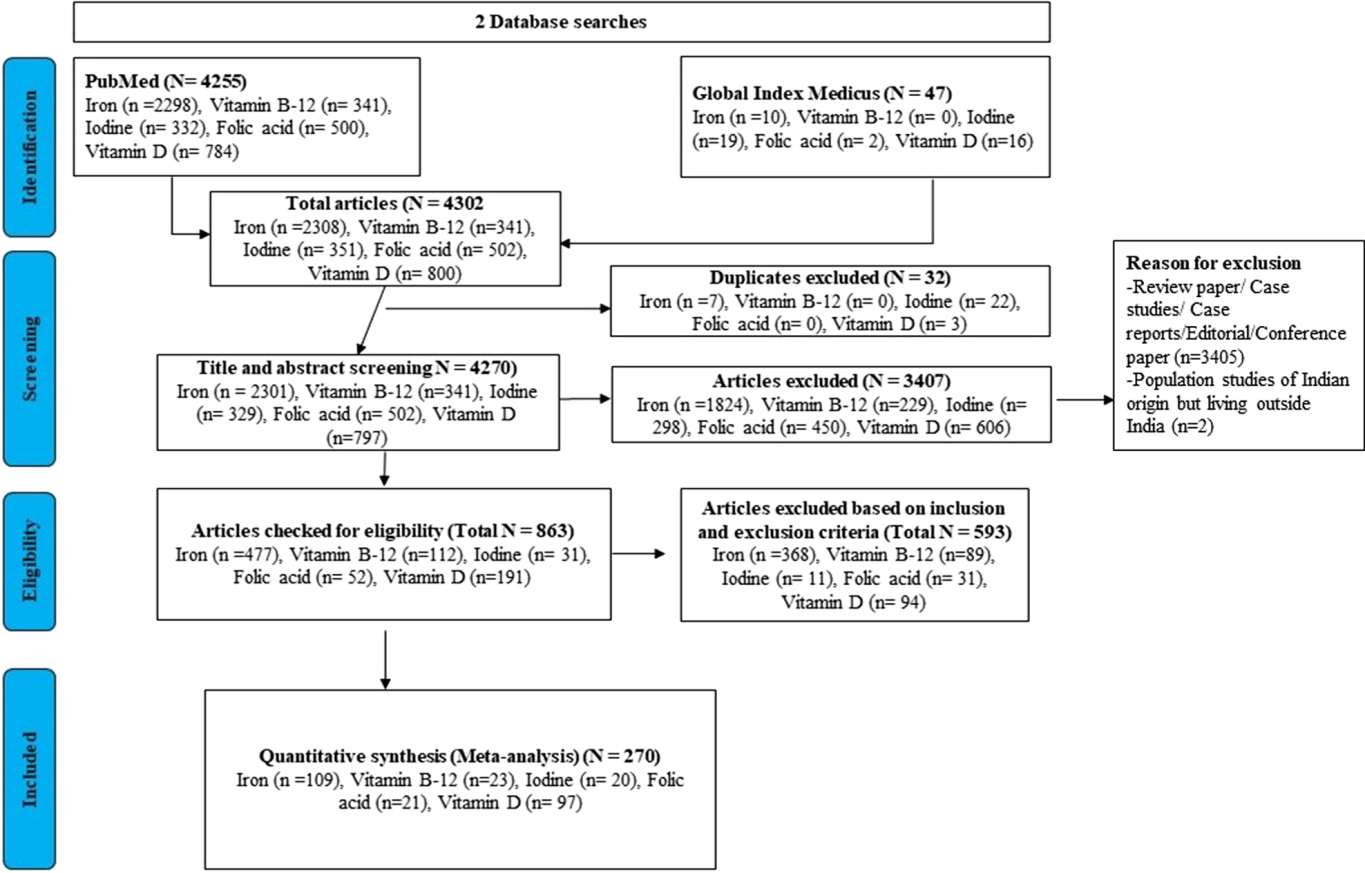
PRISMA flow diagram of studies’ screening and selection of studies for all micronutrients.

The summary of the process for selecting studies for iron, iodine, folic acid, vitamin B_12_, vitamin A and VDDs is shown in Fig. 1.

The two databases, PubMed and Global Index Medicus, were searched using Boolean keywords. A total of 4255 and 47 search results were obtained from PubMed and Global Index Medicus, respectively. Hence, the total of 4302 search results was included in the study which included 2308 (duplicate result = 7) studies regarding iron deficiency, 341 (no duplicate results) regarding vitamin B_12_, 351 (duplicate result = 22) regarding IDD, 502 (no duplicate results) regarding folic acid deficiency and 800 (duplicate result = 3) regarding VDD. Only 4270 articles were included for a title and abstract screening that further excluded 3407 articles, whereas the remaining 863 were checked for eligibility criteria and 593 articles were excluded after applying inclusion and exclusion criteria.

Finally, only 270 articles were included in the meta-analysis study which included 109 studies regarding iron deficiency, 23 regarding vitamin B_12_, 20 regarding IDD, 21 regarding folic acid deficiency and 97 regarding VDD. 270 articles qualify for the study with 50 % falling under the category of good and 50 % being fair. The characteristics of the studies identified in our systematic review are shown in Supplementary Table S1.

#### Iodine

**Fig. 2.**
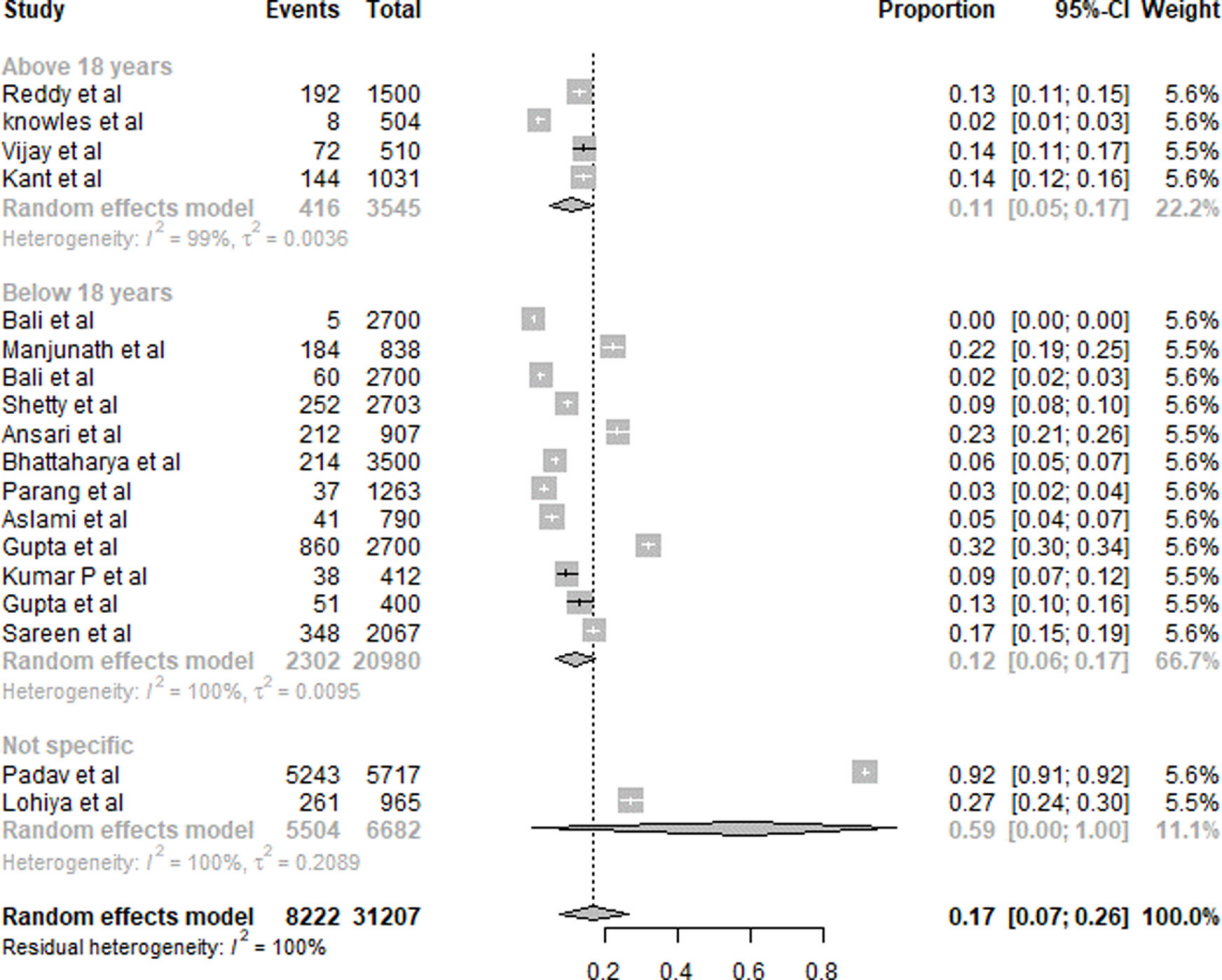
Summary of statistical analysis results of iodine deficiency among all age groups.

A summary of the results of IDD is presented in Fig. 2. Eighteen studies were included in the overall quantitative analysis. Pooled analysis from the studies shows that the overall prevalence of IDD was 17 % (95 % CI 0⋅07, 0⋅26). An 11 % (95 % CI 0⋅05, 0⋅17) prevalence of IDD was observed in subjects aged <18 years, whereas it was 12 % (95 % CI 0⋅06, 0⋅17) for subjects aged >18 years. The population with a non-specific age group was the highest with 59 % (95 % CI 0⋅00, 1⋅00).

#### Folic acid

**Fig. 3.**
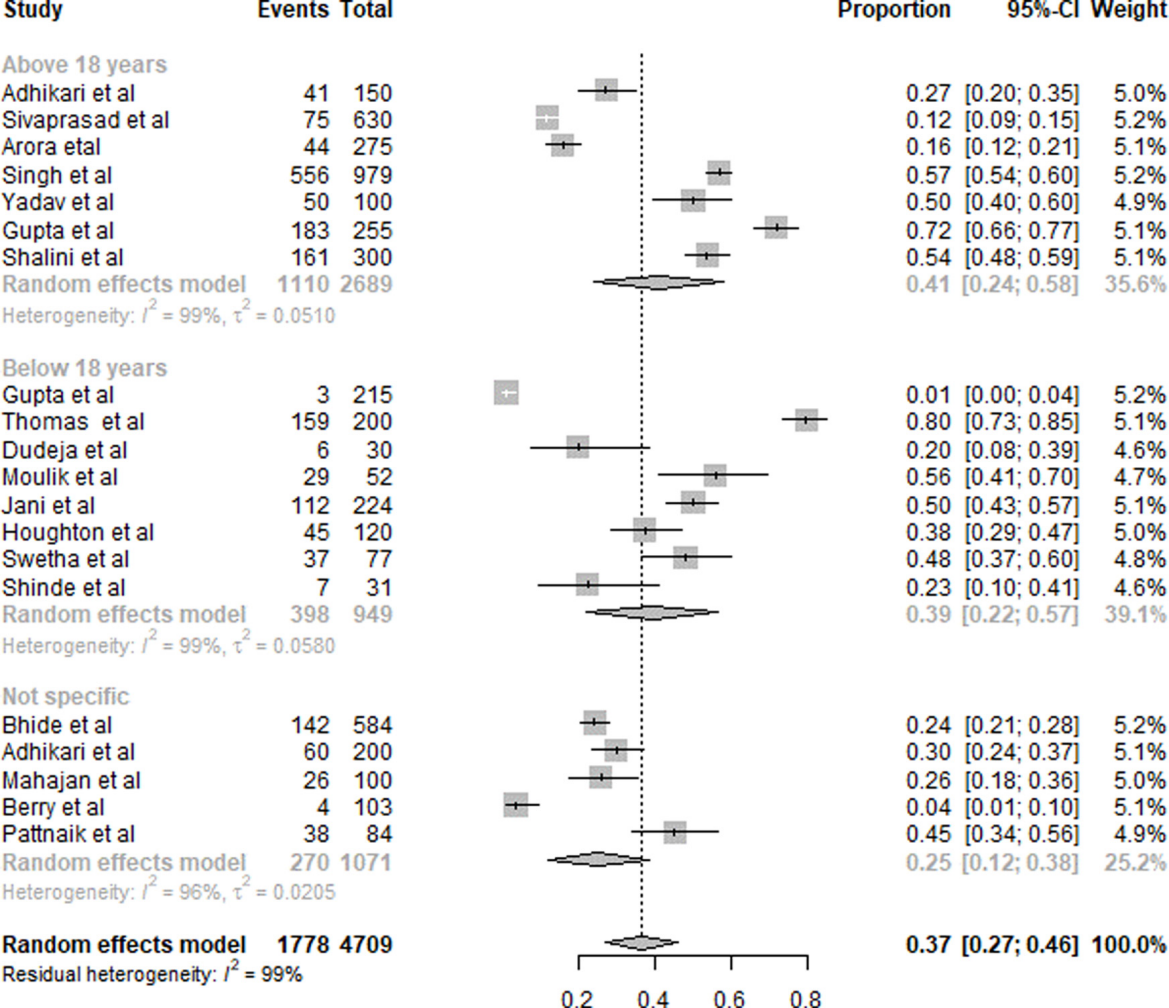
Summary of statistical analysis results of folic acid deficiency among all age groups.

A summary of folic acid deficiency results is presented in Fig. 3. Twenty studies were included in the overall quantitative analysis. Pooled analysis from the studies shows that the overall prevalence of folic acid deficiency was 37 % (95 % CI 0⋅27, 0⋅46). The prevalence of folic acid deficiency in subjects aged <18 years was 39 % (95 % CI 0⋅22, 0⋅57), and for >18 years was 41 % (95 % CI 0⋅24, 0⋅58). For the subjects with a non-specific age group, the prevalence was 25 % (95 % CI 0⋅12, 0⋅38).

#### Vitamin B_12_

**Fig. 4.**
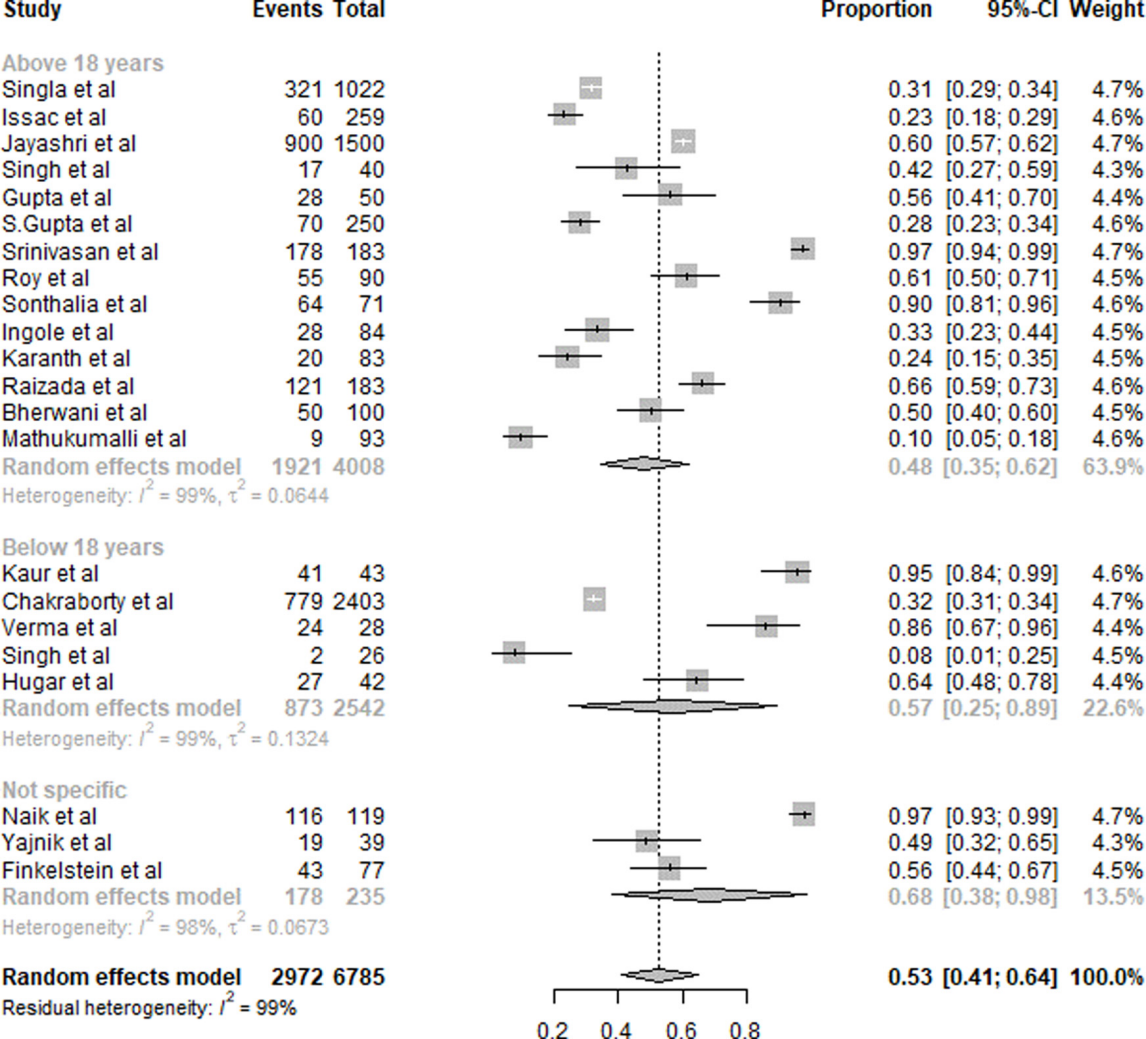
Summary of statistical analysis results of vitamin B_12_ deficiency among all age groups.

A summary of the results of B_12_ deficiency is presented in Fig. 4. Twenty-six studies were included in the overall quantitative analysis. Pooled analysis from the studies shows that the overall prevalence of B_12_ deficiency was 53 % (95 % CI 0⋅41, 0⋅64). The prevalence of vitamin B_12_ deficiency was slightly higher in subjects aged <18 years with 57 % (95 % CI 0⋅25, 0⋅89) compared to 48 % (95 % CI 0⋅35, 0⋅62) subjects aged >18 years. Due to the inclusion of only five studies in subjects aged <18 years, the CI was wider. The prevalence of subjects with non-specific age groups was 68 % (95 % CI 0⋅38, 0⋅98).

#### Vitamin A

**Fig. 5.**
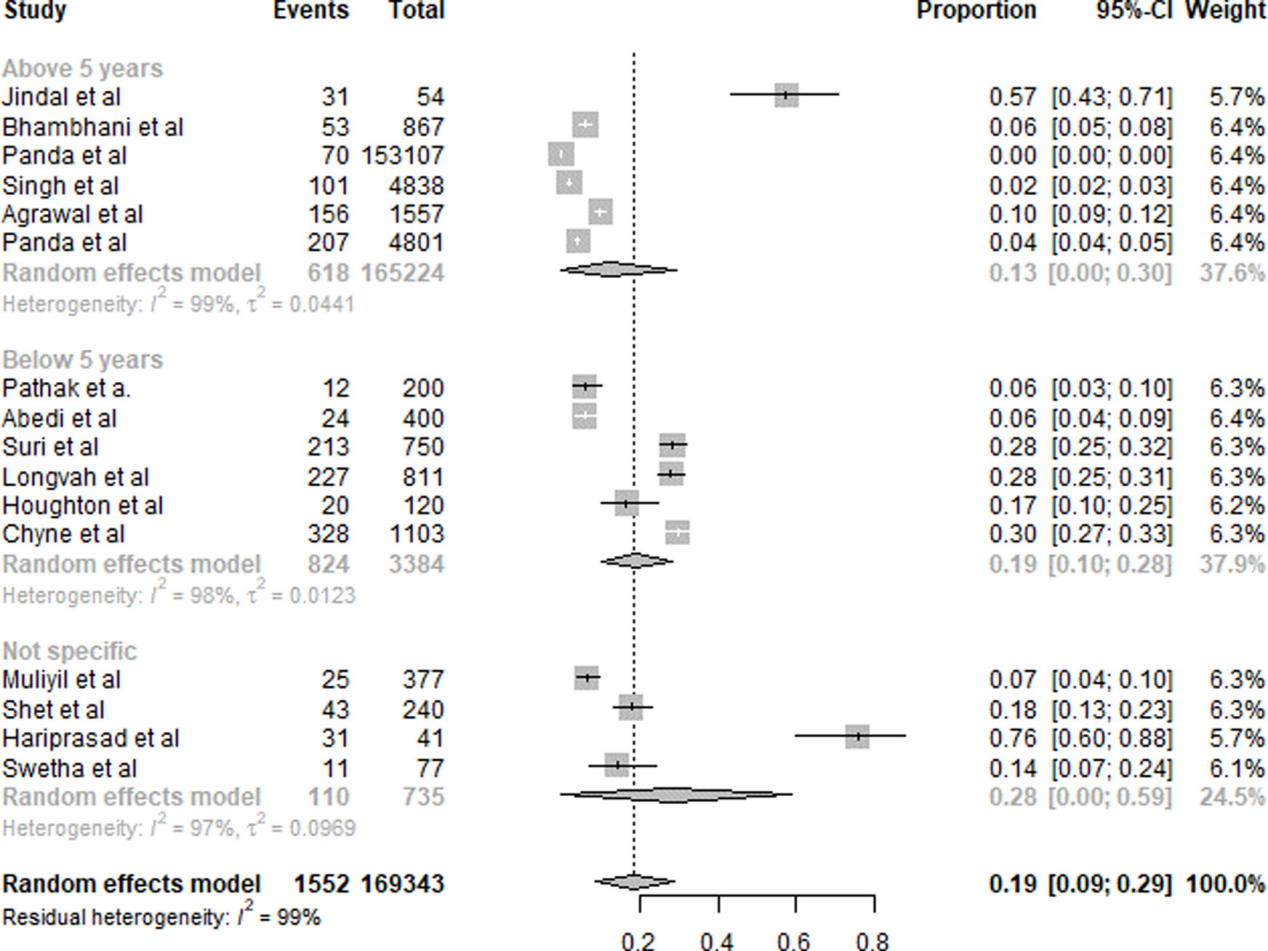
Summary of statistical analysis results of vitamin A deficiency.

A summary of the results of VAD is presented in Fig. 5. Sixteen studies were included in the overall quantitative analysis. Pooled analysis from the studies shows that the overall prevalence of VAD was 19 % (95 % CI 0⋅09, 0⋅29). The prevalence of VAD was slightly higher in subjects aged <5 years with 19 % (95 % CI 0⋅10, 0⋅28) compared to subjects >5 years with 13 % (95 % CI 0⋅00, 0⋅30). For subjects of the non-specific age group, it was 28 % (95 % CI 0⋅00, 0⋅59).

#### Iron

**Fig. 6.**
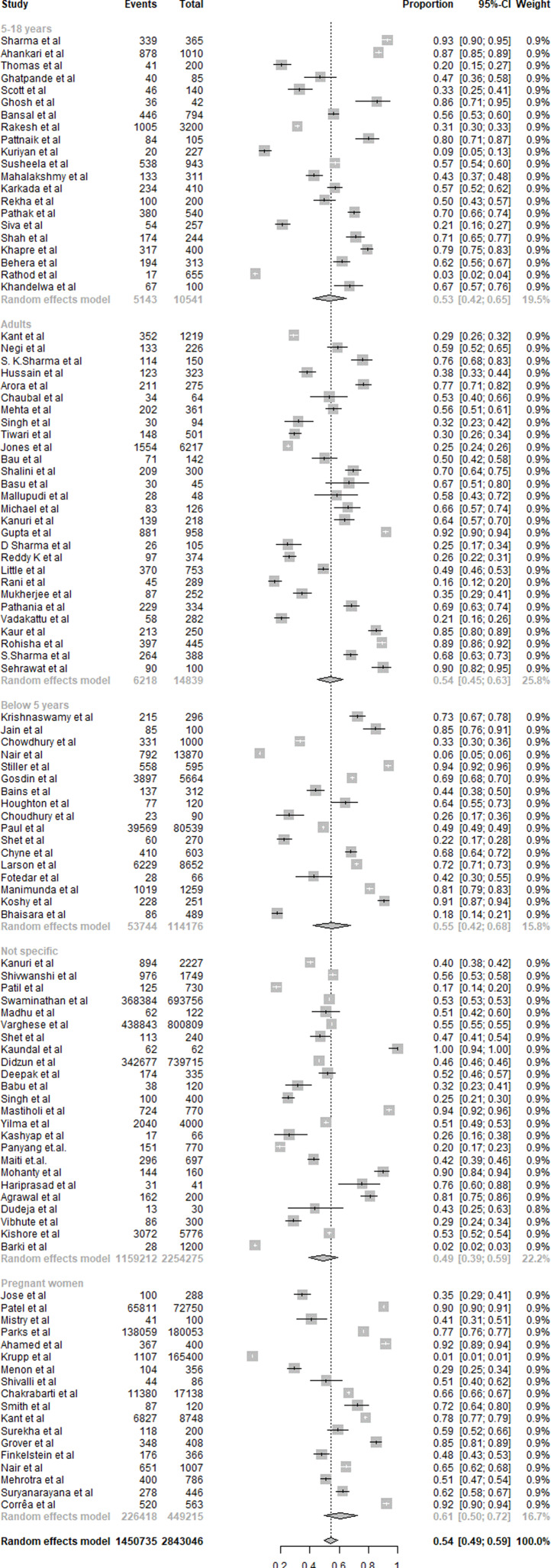
Summary of statistical analysis results of iron deficiency among all age groups.

A summary of iron deficiency results is presented in Fig. 6. One hundred eight studies were included in the overall quantitative analysis. Pooled analysis from the studies shows that the overall prevalence of iron deficiency was 54 % (95 % CI 0⋅49, 0⋅59). The prevalence of iron deficiency was the highest among pregnant women with 61 % (95 % CI 0⋅50, 0⋅72) compared to other age groups. The age group between 5 and 18 showed 53 % (95 % CI 0⋅42, 0⋅65) prevalence of iron deficiency, whereas the age group of adults and <5 years showed 54 % (95 % CI 0⋅45, 0⋅63) and 55 % (95 % CI 0⋅42, 0⋅68), respectively. The subjects with a non-specific age group showed a prevalence of 49 % (95 % CI 0⋅39, 0⋅59).

#### Vitamin D

**Fig. 7.**
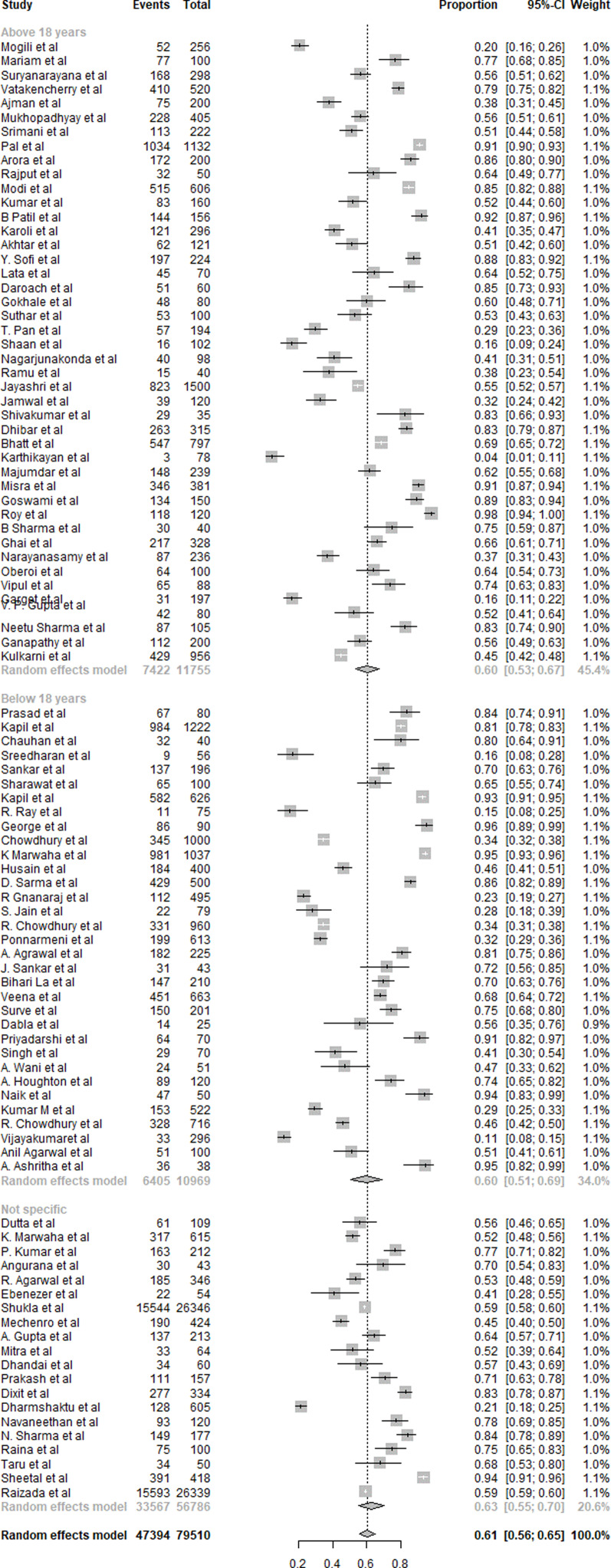
Summary of statistical analysis results of vitamin D deficiency among all age groups.

A summary of the results of VDD is presented in Fig. 7. Ninety-seven studies were included in the overall quantitative analysis. Pooled analysis from the studies shows that the overall prevalence of VDD was 61 % (95 % CI 0⋅56, 0⋅65). The prevalence of VDD was similar in subjects aged <18 years with 60 % (95 % CI 0⋅51, 0⋅69) and in those aged >18 years with 60 % (95 % CI 0⋅53, 0⋅67). Non-specific aged subjects showed 63 % (95 % CI 0⋅55, 0⋅70) prevalence of VDD. Eager's tests showed no significant publication bias (*P* > 0⋅05) for all age groups in the selected studies.

### Prevalence of micronutrient deficiencies

Table 1 shows the summary of the results of all micronutrient deficiencies prevalence in India. There was a fair distribution of studies from various age groups in the analysis of all micronutrient deficiencies. The most prevalent micronutrient deficiency was VDD, with a prevalence of 61 %. It was followed by iron deficiency of 54 %, vitamin B_12_ deficiency of 53 % and IDD of 17 %. Iron deficiency was most prevalent (61 %) in pregnant women. In the studies reporting on non-specific age groups, the higher prevalence was especially observed with deficiencies of iodine, vitamin A and vitamin B_12_ with wider CI. The current meta-analysis divulged significant differences in the prevalence of all the six micronutrients under study.

**Table 1.** Prevalence of six preventable micronutrient deficiencies included in the meta-analysis

Micronutrients	Age group	Prevalence^a^ (95 % CI)^a^
Iodine	Pooled	**17 (7, 26)**
	<18 years	11 (5, 17)
	>18 years	12 (6, 17)
	Non-specific	59 (0, 100)
Folic acid	Pooled	**37 (27, 46)**
	<18 years	39 (22, 57)
	>18 years	41 (24, 58)
	Non-specific	25 (12, 38)
Vitamin B_12_	Pooled	**53 (41, 64)**
	<18 years	57 (25, 89)
	>18 years	48 (35, 62)
	Non-specific	68 (38, 98)
Vitamin A	Pooled	**19 (9, 29)**
	<5 years	19 (10, 28)
	>5 years	13 (0, 30)
	Non-specific	28 (0, 59)
Iron	Pooled	**54 (49, 59)**
	<5 years	55 (42, 68)
	5–18 years	53 (42, 65)
	Adults	54 (45, 63)
	Pregnant women	61 (50, 72)
	Non-specific	49 (39, 59)
Vitamin D	Pooled	**61 (56, 65)**
	<18 years	60 (51, 69)
	>18 years	60 (53, 67)
	Non-specific	63 (55, 70)

a The values are expressed in percentage (%). Bold values are given for overall prevelence i.e., pooled prevelence.

Publication bias for all the six micronutrients understudy was evaluated using a Funnel plot according to age groups. Egger's test showed that the *P*-value was higher than 0⋅05 for all age groups, indicating no significant publication bias in the selected studies. Supplementary Fig. S1(a)–(e) shows the funnel plot of publication bias for iron.

## Discussion

The present meta-analysis is the first of its kind, to evaluate and analyse the preventable micronutrient deficiency in India, to the best of our knowledge. The guidelines suggest that data from at least two databases should be included in any research study^(16)^. Hence, we selected and incorporated data from the PubMed database as it is considered a reliable resource for scholarly communications services as well as from GIM^(17)^. It was observed that VDD was the most prevalent micronutrient deficiency (61 %) which was followed by iron deficiency (54 %) and vitamin B_12_ deficiency (53 %). IDD was the least prevalent with 17 %.

According to the WHO, national surveys such as NFHS-4, DLHS, NNMB and AHS have been executed to evaluate the health and status of nutrition of the population in the country. They were concentrated on dietary intake, anthropometric measurements and anaemia, whereas individualistic surveys have been executed to find various micronutrient deficiencies^(1)^. According to the Vitamin and Mineral Nutrition Information System (VMNIS-2005), globally, anaemia affects 1⋅62 billion people (95 % CI 1⋅50, 1⋅74 billion), which corresponds to 24⋅8 % of the population (95 % CI 22⋅9, 26⋅7 %)^(18)^. In the present analysis, it was found that iron deficiency is 54 % that is more than the VMNIS reported results.

A recent survey executed by the WHO and the Ministry of Health and Family Welfare presented with the report that the prevalence of anaemia was 58⋅6, 53⋅1, 50⋅4 and 22⋅7 %, among children aged 6–59 months, women aged 15–49 years, pregnant women aged 15–49 years and men aged 15–49 years, respectively^(1,2,4)^. The Indian Council of Medical Research (ICMR), New Delhi carried out a study over sixteen districts of eleven states that included 11 260 pregnant women with 6923 study subjects, and 4337 adolescent girls also presented the report with the prevalence of anaemia of 84⋅9 and 90⋅1 %, respectively^(19)^. The present analysis provides information that children under 18 years are majorly suffering from different vitamin deficiencies and iron deficiency was highly prevalent in pregnant women. We noticed that in several studies, micronutrient deficiency was improved from taking respective supplements for a particular vitamin deficiency^(20–25)^. This information enables us to think about the fortification of food with vitamin supplements to treat such deficiencies.

Vitamin B_12_ deficiency is believed to be widespread in India. According to the previous study performed in 2019, 47 % of the north Indian population is deficient in vitamin B_12_ which included a higher percentage of diabetic patients^(26)^. Another community-based study from south India showed that vitamin B_12_ deficiency (55 %) was prevalent in pregnant women belonging to rural India^(27)^. In the present study, we discovered that the prevalence of vitamin B_12_ deficiency is 53 % which is almost similar to previous studies from various parts of India.

VAD is also an obstinate problem faced by the Indian population. A study reported that the scenario is worst in the case of pre-school children (62 %) in South Asian countries and India is at the topmost position for the same among other countries^(28)^. A study done on Southern Indian Tribal Population reported that among the children (1–8 years), the prevalence of VAD was 10⋅2 %^(29)^. Nearly half of the pre-school children were affected by severe VAD in South Asian regions, according to the WHO^(4)^. The pooled analysis from the present review revealed a prevalence of VAD of 19 % in children aged <5 years which is almost double compared to the study done in the south Indian tribal population^(4)^. The prevalence of VAD could be affected by several factors such as food habits, socio-economic factors, healthcare availability, the commitment of primary healthcare workers and the community's participation. The cut-off levels of serum retinol levels and operational definitions used could have also been the cause of this varied prevalence across studies. The prevalence of night blindness in pre-school children is negligible in Europe (1 %) and almost nil in America^(4)^.

UNICEF in 2006 estimated that 13 million newborns were unprotected against IDD disorders in India as only 51 % of households were consuming adequately iodised salt^(30)^. In our analysis, we observed a 17 % prevalence of IDD in the Indian population. In another study, among rural children of eight states, the prevalence of IDD measured by goitre prevalence was 3⋅9 %^(12)^. This lower prevalence in their study could be due to the children of school age only being included in their study. Universal salt iodisation may be the most optimistic, enduring and economical solution to refrain from the onerous problem of IDD at the country level^(31)^.

The WHO has reported 41⋅6 % of pregnant women from India to show a serum folic acid level <3 ng/ml. Disturbed serum folic acid levels during pregnancy can result in various unwanted outcomes related to the health of the mother and infants like preeclampsia, preterm delivery, congenital heart defects, abruptio placentae, low birth weight, spontaneous abortion, stillbirth and serious neural birth defects^(32)^. The present study showed similar results with the 37 % prevalence of folic acid deficiency among the Indian population.

VDD is commonly underdiagnosed and undertreated in most parts of the world, as patients do not present with apparent clinical symptoms till there is a severe deficiency^(33,34)^. The skeletal complications of VDD like rickets, osteomalacia and osteoporosis are well known in India. The prevalence of VDD ranged from 50 to 94 % in various age groups^(35)^. These community-based studies most commonly used 25-hydroxy vitamin D levels for diagnosis. The prevalence of VDD was 56⋅3 % in the urban elderly^(36)^. The overall pooled prevalence of 61 % in the present meta-analysis is in accordance with the prevalence of VDD reported across various age groups^(35–37)^. More than half of the population in each age group is suffering from VDD in India. Irrespective of the age groups (<18 or >18 years), VDD has become rampant in India. In a review on hypovitaminosis D in India, the prevalence ranged from 84⋅9 to 100 % in children of school-going age, 42–74 % in pregnant women, 44⋅3–66⋅7 % in infants and 30–91⋅2 % in adults^(37)^. In India, VDD is quite rampant. Reduced diet intake, food practices, cultural practices like purdah, inadequate exposure to sunlight due to urbanisation, pollution and low-income, family spacing are some of the responsible factors. Milk and edible oil are being fortified with vitamin D^(21)^. It is preferable to provide calcium supplementation also along with vitamin D. The fortification of milk with vitamin D has been found to be efficient. The population through health education can go a long way in combating these deficiencies.

The use of fortified foods can address the problem of micronutrient deficiency. Enrichment of food is the process of adding micronutrients to food. The routinely consumed foods that are suitable for an economically weak population of the country are identified and chosen for fortification. The fortification of food with solo or dual micronutrients greatly impacts the population of children and women suffering from micronutrient deficiency. Several studies have concluded that the fortification of food with iron, zinc and multiple-micronutrient supplementation has shown a considerable effect in children and women^(38)^. Currently, in India, there are various single and double fortified staples with iron, folic acid, vitamin B_12_ that include wheat flour and rice^(14)^. Currently, the fortified food market can provide double fortified salt fortified with iron and iodine, and lipid-based nutritional supplements like edible oil and milk are fortified with vitamin A that reduces anaemia and stunting and underweight by improving nutritional outcomes^(39)^.

The fortified processed products are generally safe. But excess addition of vitamins and minerals can also impose serious risks. Excess vitamin A intake can reduce bone density, increase birth defect risks and cause liver damage^(40)^. The fortification of foods with vitamins and minerals may result in decreased absorption, failure of treatment, increased mortality risk and harmful interactions with people taking other prescription medications^(41)^. Hence, awareness about the optimal intake of fortified foods is necessary. Also, poor nutrition cannot be overcome by food fortification alone.

Besides food fortification, other evidence-based interventions such as dietary diversification, nutritional education, micronutrient supplementation and environmental sanitation and hygiene are other measures taken to tackle micronutrient malnutrition^(31)^. The consumption of a variety of food rich in vitamin A and iron through dietary diversification has gained popularity in India to combat micronutrient deficiencies. Health and nutrition education is needed for long-term sustainable results to control and prevent micronutrient deficiencies. These measures also need the involvement of multiple sectors besides the Department of Health and Family Welfare.

The present study may assist the food fortification system in India, as we have provided an age-wise prevalence of micronutrient deficiency. Also, a meta-analysis of data that are collected from various parts of India describes the genuine requirement of fortification of micronutrients peculiarly iron, iodine, folic acid, vitamin A, vitamin D and vitamin B_12_. Some more serious meta-analysis is required to get more idea about the pattern of micronutrient deficiencies in India. This kind of analysis will turn out to be very efficient in preparing fortified staple foods and providing them to the actual sufferers of this horrific micronutrient deficiency. Especially effectiveness of meta-analysis should support and spread in countries and must reach more targeted populations.

This is the first meta-analysis that includes the prevalence of micronutrient deficiencies of vitamin A, vitamin B_12_, vitamin D, iron, iodine and folic acid altogether. The present study was endeavoured with substantialness, including its concise nature and consideration of the effectiveness of studies. The protocol was registered prospectively. We tried to collect all relevant studies that help us in decision-making from large-scale national surveys as well as small studies over the last 5 years to estimate the prevalence of anaemia in India. Along with the solidity, there are few shortcomings of the present study like we have included data from two databases only. The main limitation of this analysis is that it does not provide the micronutrient deficiency data in every part of India but it gives an approximate idea about micronutrient deficiencies in India. Also, the present study lacks a gender-wise analysis of data. The effect of micronutrient deficiency in women is usually more severe than in men because of increased requirements at various stages of their life. Its implications include higher prevalence of anaemia in pregnant women and its effects on foetus and mother, association of vitamin D levels with infertility and Poly Cystic Ovarian Disease in women, congenital anomalies and effects on the foetus.

## Conclusion

The most prevalent micronutrient deficiency was VDD (61 %), followed by iron (54 %), vitamin B_12_ deficiency (53 %), folic acid (37 %) and IDD 17 %. With regard to studies reporting on non-specific age groups, the higher prevalence was observed with iodine, vitamin A and vitamin B_12_ deficiencies. The results of the present study are intended to reinforce the data on micronutrient deficiency in Indian and may assist in food fortification. As VDD was prevalent among the population, there is the need to initiate proven public health interventions such as the fortification of foods to address the deficiencies. Although fortification can be promising, it may not be the only answer to the widespread nutritional deficiencies and may need the help of other interventions besides innovative and region-specific strategies.

## Supplementary material

For supplementary material accompanying this paper visit https://doi.org/10.1017/jns.2021.102. click here to view supplementary material
