# Supplementary material for: Micronutrient's deficiency in India: a systematic review and meta-analysis
Source: J Nutr Sci. 2021 Dec 21;10:e110. doi: 10.1017/jns.2021.102 (PMC8727714; doi:10.1017/jns.2021.102)
Supplement: Supplementary file 1 [file jnssup.zip › S2048679021001026sup001.docx]

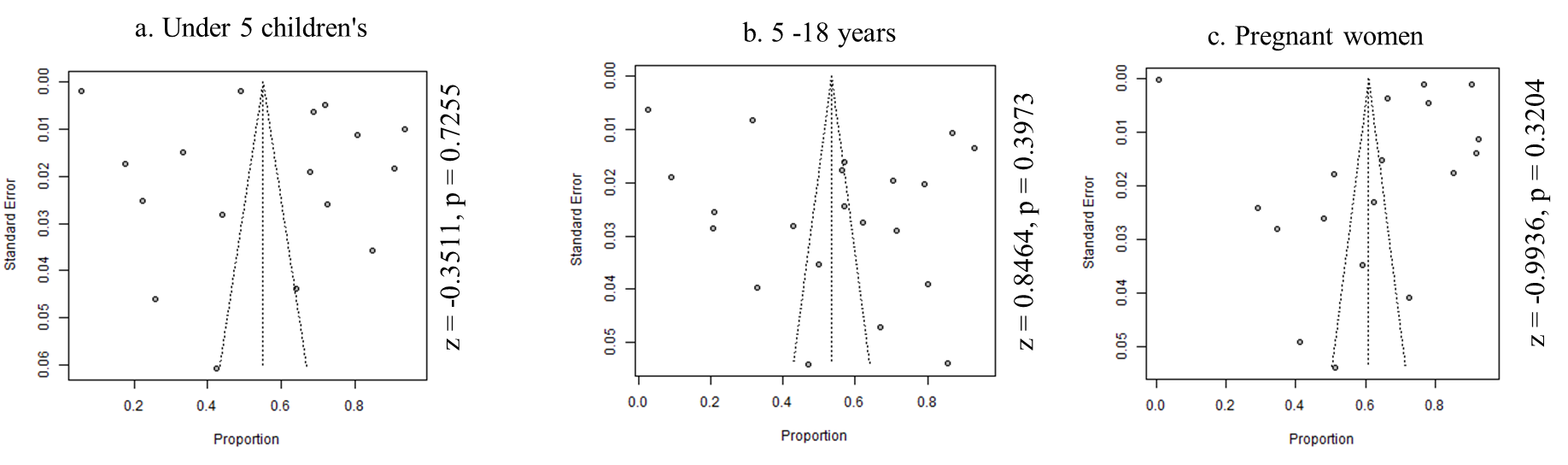

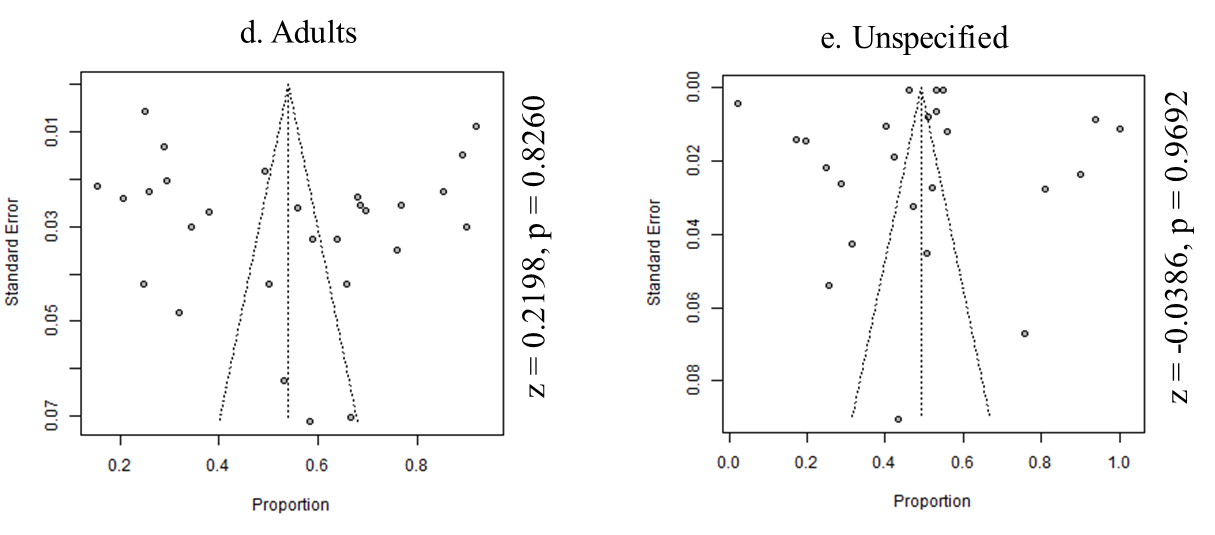


**Supplementary Figure S1:** Funnel plot of studies included in the Iron micronutrient deficiencies among different age groups (a) Under 5 children’s (b) 5-18 years (c) Pregnant women (d) Adults (e) Unspecified
