# Supplementary material for: Micronutrient's deficiency in India: a systematic review and meta-analysis
Source: J Nutr Sci. 2021 Dec 21;10:e110. doi: 10.1017/jns.2021.102 (PMC8727714; doi:10.1017/jns.2021.102)
Supplement: Supplementary file 1 [file jnssup.zip › S2048679021001026sup002.docx]

| **Table S1: Characteristics of the studies identified in the systematic review of the prevalence of micronutrient deficiency among people in India.** | | | | | | | | | | | | | | |
| --- | --- | --- | --- | --- | --- | --- | --- | --- | --- | --- | --- | --- | --- | --- |
| **Author** | **Year of publication** | **Year of study** | **Place of study** | **Study design** | **Study setting** | **Sampling technique** | **Study population** | **Age group of SPs (yrs)** | **Scales used** | **Sample size** | **Micronutrient deficient samples** | **Prevalence** | **95 CI%** | **Determinants/Risk factors** |
| T Saravana Kumar Reddy et al | 2019 | Aug2015-jul17 | Udupi, Karnataka | Cross-sectional | Community | Stratified | Women of reproductive age group | 18-49 | Grades as per WHO | 1500 | 192 | 13% | 0.85-1.79 | Consumption of iodized salt |
| Surya Bali et al | 2019 | Jun-Jul 2016 | Tikamgarh, Madhya Paradesh | Cross-sectional | Community | Cluster | School children | 6-12 | Grades 1 and 2 | 2700 | 5 | 0.2% |  | Iodized salt intake |
| Bhanu Manjunath et al | 2015 | 2015 | Rural Karnataka | Cross-sectional | Community | Simple random | School children | 6-12 | WHO goiter survey | 838 | 184 | 21.9% | 19.2-24.8 | Low iodized salt intake |
| Jacjy Knowles et al | 2018 | Dec2014-Apr 2015 | India | Cross-sectional | Community | Stratified | Women of reproductive age group | 18-49 | Multidimensional poverty index | 504 | 8 | 1.6% | 1.3-2 | Non iodized salt |
| Jyoti Vijay et al | 2018 | Jan- Mar 2015 | Tonk, Rajasthan | Cross-sectional | Institutional | Random | Pregnant mothers |  | TGR grades | 510 | 72 | 14.2% | 11.2-17.2 | Iodized salt intake, socio economic status |
| Surya Bali et al | 2019 | 2017 | Jabalpur, India | Cross-sectional | Community | Cluster | School children | 6-12 | WHO grades | 2700 | 60 | 2.2% |  | Iodized salt intake |
| Jacjy knowles et al | 2018 | 2014-15 | 6 Zones of India | Cross-sectional | Community | Stratified | Women of reproductive age group | 15-49 | MPI and WHO grades | 504 | 33 | 6.5% | 4.9,8.8 | Iodized salt, socio economic status |
| ChandrakanT S Padav et al | 2018 | Sep 2014-Aug15 | 6 Zones of India | Cross-sectional | Community | Stratified | Women/adult | - | MPI and survey | 5717 | 5243 | 91.7% | 91.0,92.7 | Endemic goiter |
| Avinash Shetty et al | 2018 | 2016 | Udupi, Karnataka | Cross-sectional | Institutional | Random | School children | 6-12 | Grades as per WHO /UNICeF | 2703 | 252 | 9.3% |  | Goiter prevalence |
| Mohammed Ansari et al | 2017 | 2015 | Aligarh | Cross-sectional | Institutional | Random | School children | 6-12 | Statistical analysis | 907 | 212 | 23.3% |  | Endemic goiter |
| Udayan Bhattaharya et al | 2019 | 2016 | Kolkata, West Bengal | Cross-sectional | Institutional | Cluster | School children | 6-12 | Median urinary iodine level 21.80 micro gram /dl | 3500 | 214 | 6.1% | 22.16-25.19 | Inadequate iodized salt consumption |
| Kislay Parang et al | 2018 | 2015 | Bihar | Cross-sectional | Community | Cluster | Children | 6-12 | TGR and IQ | 1263  HH | 37 | 2.9% |  | Iodized salt intake |
| Ahmad Nadem Aslami et al | 2016 | 2015 | Aligarh | Cross-sectional | Hospital | Cluster | School children | 6-12 | Median urinary iodine excretion | 790 | 41 | 5.2% |  | Hormonal imbalance, stress, strain |
| Vithal D. Udagatti et al | 2017 | June 2014 - February 2015 | Raichur | Observational study | Hospital |  |  | All age |  | 100 | Iodine | 26% |  |  |
| Shashi Kant et al | 2017 | March to May 2015 | Ballabgarh, District Faridabad, Haryana | Cross-sectional | Hospital | Consecutive sampling |  | 18–30 |  | 1031 | Iodine | 14% |  |  |
| Puneet Gupta et al | 2020 | 2014-2015 | Delhi | Cross-sectional | Hospital |  | Children | 6-14 |  | 2700 | 172 | 6.40% |  |  |
| Kumar P et al | 2019 | 2017 | Begusarai, Bihar | Cross-sectional | Community | Cluster | Children | 6-12 | MBI kit, titration | 412 household | 38 | 9.3% |  | Iodized salt, thyroid goiter |
| Lohiya et al | 2015 | 2015 | Faridabad, Haryana | Cross-sectional | Community | Random | Adults |  | MIC microplate method | 965 | 261 | 27% |  | Iodized salt, |
| Gupta et al | 2015 | 2015 | Lucknow | Cross-sectional | Community | Random | School children | 6-12 |  | 400 |  | 12.7% |  | Iodized salt, |
| Sareen et al | 2015 | 2015 | Pauri, Uttarakhand | Cross-sectional | Clinical | Cluster | School children | 6-12 | Median UIC level | 2067 | 348 | 16.8% |  | Iodized salt, remote area |

| **Table 2: Characteristics of the studies identified in the systematic review of the prevalence of Vitamin B 12 deficiency among people in India.** | | | | | | | | | | | | | | |
| --- | --- | --- | --- | --- | --- | --- | --- | --- | --- | --- | --- | --- | --- | --- |
| **Author** | **Year of publication** | **Year of study** | **Place of study** | **Study design** | **Study setting** | **Sampling technique** | **Study population** | **Age group of SP (Yrs)** | **Scales used** | **Sample size** | **Micronutrient deficient samples** | **Prevalence** | **95 CI%** | **Determinants/Risk factors** |
| [Sadanand Naik](https://pubmed.ncbi.nlm.nih.gov/?term=Naik+S&cauthor_id=29446340) et al | 2018 | 2015 | Pune | Experimental study | Hospital based | Random | Vegetarian peoples |  | Buderer’s method | 119 | 116 | 97.48 | 92.85-  99.14 | Neurological symptoms, Hyperhomocysteinaemia |
| [Rajiv Singla](https://pubmed.ncbi.nlm.nih.gov/?term=Singla+R&cauthor_id=31161105) et al | 2019 | 2016-2018 | Haryana | Retrospective and cross-sectional study | Community | Random | Younger aged diabetics peoples | >18 | Electrochemiluminescence assay | 1022 | 321 | 31.41 | 28.64-  34.32 | Neuropathic symptoms |
| [Thomas Gregor Issac](https://pubmed.ncbi.nlm.nih.gov/?term=Issac+TG&cauthor_id=25722508) et al | 2015 | 2014 | South India | Retrospective study | Community | Mixed | Elderly | >60 | Anemia | 259 | 60 | 23.17 | 18.45-  28.68 | Neuropsychiatric illnesses, Helicobacter pylori infection |
| [Sukhjot Kaur](https://onlinelibrary.wiley.com/action/doSearch?ContribAuthorStored=Kaur%2C+Sukhjot) et al | 2018 | 2010-2017 | Punjab | Retrospective study | Community | Mixed | Skin changes of vitamin B12 deficiency in infants | 4-27 months | Anemia | 43 | 41 | 95.35 | 84.55-  98.72 | Dermatologic manifestations, cutaneous manifestations |
| [Sandeep Tak](https://pubmed.ncbi.nlm.nih.gov/?term=Tak+S&cauthor_id=31321933) et al | 2018 |  |  | Prospective observational study | Hospital based | Mixed | Dengue fever patient | 25+12 | Standard statistical method | 40 | 21 | 52.5 | 37.5-  67.06 | Bleeding, serositis, shock, severe thrombocytopenia |
| Ramamoorthy Jayashri et al | 2018 | 2001-2003 2012-2013 | Chennai | Cohort study | Community | Random | Urban south Indian population | <20 - >80 | Anthropometric | 1500 | 900 | 60 | 57.5-  62.45 | Severity of glucose tolerance, chronic pancreatitis |
| Charu Singh et al | 2016 | 2012-2013 | Lucknow | Placebo-controlled prospective study | Community | Random | Adult patients | 18-60 | Neurophysiologic model of Jastreboff | 40 | 17 | 42.5 | 28.51-  57.8 | Weak neural activity in the periphery, Chronic tinnitus |
| [S Chakraborty](https://pubmed.ncbi.nlm.nih.gov/?term=Chakraborty+S&cauthor_id=29468754) et al | 2018 |  | Haryana | Cross-sectional study | Community | Mixed | Healthy Indian school-going adolescents | 11-17 | Electrochemiluminescence immunoassay | 2403 | 1556 | 64.75 | 62.82-  66.64 | Anthropometric indices, Adiposity indices |
| [Kamesh Gupta](https://pubmed.ncbi.nlm.nih.gov/?term=Gupta+K&cauthor_id=28882470) et al | 2018 | 2014-2016 | New-Delhi | Observational study | Hospital based | Mixed | Type 2 DM patients | <18 | Electrochemiluminescence immunoassay method | 50 | 28 | 56 | 42.31-  68.84 | Coalmine deficiency, Gastro-Intestinal surgery |
| [Ekant Surendra Gupta](https://pubmed.ncbi.nlm.nih.gov/?term=Gupta+ES&cauthor_id=27437269) et al | 2016 | 2013-2014 | Western India | Cross-sectional study | Hospital based | Mixed | RO processed water for drinking | >18 | Anemia | 250 | 70 | 28 | 22.8-  33.87 | Cardiovascular morbidity and mortality |
| [KrishnamaChari Srinivasan](https://pubmed.ncbi.nlm.nih.gov/?term=Srinivasan+K&cauthor_id=27356547) et al | 2017 | 2007-2014 | Bangalore | Placebo-controlled randomized study | Community | Random | <14 weeks of gestation | <14 weeks- 9 months | Anemia | 183 | 178 | 97.27 | 93.77-  98.83 | Diabetes mellitus, hypertension, heart disease or thyroid disease |
| [Deepti Verma](https://pubmed.ncbi.nlm.nih.gov/?term=Verma+D&cauthor_id=28667789) et al | 2017 |  | New-Delhi | Prospective observational study | Community | Mixed | Children | 6 months-18 yrs | Anemia, oral methyl cobalamin therapy | 28 | 24 | 85.71 | 68.51-  94.3 | Macrocytic anemia |
| [Chittaranjan S Yajnik](https://pubmed.ncbi.nlm.nih.gov/?term=Yajnik+CS&cauthor_id=31600243) et al | 2019 |  | Pune |  | Community | Random | B-12 deficient rural Indian adolescent women |  | Anemia | 39 | 19 | 48.72 | 33.87-  63.8 | Pernicious anemia |
| [Rudra Prasad Roy](https://pubmed.ncbi.nlm.nih.gov/?term=Roy+RP&cauthor_id=27730072) et al | 2016 | 2010-2012 | Kolkata | Cross-sectional study | Hospital based | Random | Diabetic patients | 35-70 | Oral Hypoglycemic Agent (OHA) treatment | 90 | 55 | 61.11 | 50.78-  70.53 | Cardiovascular diseases |
| [J L Finkelstein](https://pubmed.ncbi.nlm.nih.gov/?term=Finkelstein+JL&cauthor_id=28402324) et al | 2017 |  | Bangalore | Prospective study | Community | Random | Pregnant women | ⩽14 weeks of gestation | Vitamin B12 biomarkers | 77 | 43 | 55.84 | 44.73-  66.39 | Adverse perinatal outcomes |
| [Manjeet Singh](https://pubmed.ncbi.nlm.nih.gov/?term=Singh+M&cauthor_id=32435301) et al | 2020 | 2016-2018 | North India | Retrospective study, Cross sectional study | Hospital based | Random | Children treated for West syndrome | 6 months | Clinical treatment | 26 | 2 | 7.69 | 2.13-  24.14 | West syndrome |
| [Sidharth Sonthalia](https://pubmed.ncbi.nlm.nih.gov/?term=Sonthalia+S&cauthor_id=28584374) et al | 2017 | 2012-2015 | Gurugram | Retrospective study | Hospital based | Random | Premature canities | 25 | Anemia | 71 | 64 | 90.14 | 81.02-  95.14 | Hypothyroidism |
| [Jitendra Rajendra Ingole](https://pubmed.ncbi.nlm.nih.gov/?term=Ingole+JR&cauthor_id=26816929) et al | 2015 | April 2013- July 2013 | Pune | Observational study | Community | Mixed | IT professionals | >32 | Anemia | 84 | 28 | 33.33 | 24.17-  43.94 | Lack of exercise, diet |
| [Veena K L Karanth](https://pubmed.ncbi.nlm.nih.gov/?term=Karanth+VK&cauthor_id=26652288) et al | 2015 | July Aug 2013 | Manipal | Observer-blind study | Hospital based | Mixed | Karanth's test | 20-77 | Chemiluminescence technique | 83 | 20 | 24.1 | 16.18-  34.31 | Megaloblastic anemia |
| [Nishant Raizada](https://pubmed.ncbi.nlm.nih.gov/?term=Raizada+N&cauthor_id=28553599) et al | 2017 | 2014-2015 |  | Observational study | Hospital based | Mixed | Type 2 Diabetic | 49.8 ± 10.2 | Chemiluminescent enzyme immunoassay | 183 | 121 | 66.12 | 58.99-  72.58 | Anemia |
| [Shivayogi M Hugar](https://pubmed.ncbi.nlm.nih.gov/?term=M+Hugar+S&cauthor_id=28890613) et al | 2017 |  |  | Observational study | Hospital based | Random | Dental caries patient | 10-14 | Karl Pearson's correlation test | 42 | 27 | 64.29 | 49.17-  77.01 | Megaloblastic anemia, neurological manifestations |
| [Sonny Bherwani](https://pubmed.ncbi.nlm.nih.gov/?term=Bherwani+S&cauthor_id=28283394) et al | 2017 | 2011-2015 | New-Delhi | Cross-sectional study, case control | Hospital based | Mixed | Diabetic patients | >50 | Chemiluminescence immunoassay. | 100 | 50 | 50 | 40.38-  59.62 | Chronic metabolic disorder |
| [Neeharika L Mathukumalli](https://pubmed.ncbi.nlm.nih.gov/?term=Mathukumalli+NL&cauthor_id=32055122) et al | 2020 | 2012-2014 | Hyderabad, Telangana | Observational study | Community | Random | Hyperhomocysteinemia in patients | >60 | Chemiluminescence immunoassay | 93 | 7 | 7.53 | 3.7-  14.73 | Large-fiber axonal neuropathy |

| **Table 3: Characteristics of the studies identified in the systematic review of the prevalence of Iron deficiency among people in India.** | | | | | | | | | | | | | | |
| --- | --- | --- | --- | --- | --- | --- | --- | --- | --- | --- | --- | --- | --- | --- |
| **Author** | **Year of publication** | **Year of study** | **Place of study** | **Study design** | **Study setting** | **Sampling technique** | **Study population** | **Age group of SP (Yrs)** | **Scales used** | **Sample size** | **Micronutrient deficient samples** | **Prevalence** | **95 CI%** | **Determinants/Risk factors** |
| [Ambily Jose et al](https://www.ncbi.nlm.nih.gov/pubmed/?term=Jose%20A%5bAuthor%5d&cauthor=true&cauthor_uid=30717690) | 2019 | (Jan 2016–Aug 2017) | New Delhi | Community based | Tertiary hospital | Random sampling | Pregnant women | Not reported | Hb > 60 g/L and < 100 g/L and iron deficiency anemia | 288 | 100 | 34.72 | 29.46-  40.39 | Not reported |
| [Giridhar Kanuri et al](https://www.ncbi.nlm.nih.gov/pubmed/?term=Kanuri%20G%5bAuthor%5d&cauthor=true&cauthor_uid=30093400) | 2018 | Not reported | India | Institution based cross sectional study | Rural Indian community | Random sampling | Children and women | Not reported | Hb≥11g/dL for children and ≥12g/dL for women | 2227 | 894 | 40.14 | 38.12-  42.19 | Absent body iron stores (serum ferritin <12ng/ml |
| Shashi Kant et al | 2019 | Aug-Dec (2016) | Haryana | Community based cross sectional study | Community | Multistage simple random sampling | Adult males | ≥18 | HemoCue method (<11 g/dL) | 1219 | 352 | 28.8 | 26.41-  31.49 | Poor purchasing capacity |
| Archana Patel et al | 2018 | June 2009-December 2016 | Maharashtra | Prospective observational cohort study | Primary Health Centers | Purposive sampling | Pregnant women | Not reported | Sahli's method (Hb level <11 g/dL) | 72,750 | 65,811 | 90.46 | 90.24-  90.67 | Anaemia and low BMI/ neonatal death and stillbirth |
| P.C. Negi et al | 2018 | June 2016-dec 2017 | Himachal Pradesh | Prospective study | Tertiary hospital | Stratified sampling | Middle aged population | 58 | iron deficiency (ferritin level <100 μg/L) & anaemi-a (Hb -level<13 gm/dl) | 226 | 81 | 35.80 | 52.2-  65.1 | Low income/advanced heart failures |
| Sudarsan Krishnaswamy et al | 2017 | Jan 2013- Dec er 2014 | Chandigarh | Cross sectional study | Tertiary hospital | Simple random sampling | Infants | 3-5 months | ID (<12 μg/L) IDA (Hb ≤ 10.5 g/dl) | 296 | 215 | 72 | 67-  77 | Neurocognitive and behavioral deficits |
| Urvashi Sharma et al | 2019 | Oct 2015 – Mar 2016 | Uttar Pradesh | Not reported | Rural schools | Multistage stratified random sampling | Children | 4-6 | (Hb) level <11 g/dL | 365 | 339 | 92.88 | 89.77-  95.1 | Poor nutritional status, rural settlement |
| Lohit Raj Shivwanshi et al | 2019 | Not reported | Chhattisgarh | Not reported | Districts | Random sampling | Not reported | Not reported | Not reported | 1749 | 976 | 55.8 | 53.46-  58.11 | Sickle cell anemia |
| Ritesh Mistry et al | 2018 | Apr (2015-2016) | Mumbai | Observational study | Clinic patients | Purposive sampling | Pregnant women | 18-45 | HemoCue method | 100 | 72 | 72 | 62.51-  79.86 | Consumption of iron rich foods, food insecurity |
| Prashant Patil et al | 2019 | Jan 2016- Aug 2017 |  | Randomized controlled trial | Tertiary hospital | Stratified random sampling | Children | 1-12 | Hb< 10(g%) | 730 | 125 | 17.12 | 14.56-  20.02 | Not reported |
| Sumathi Swaminathan et al | 2019 | Not reported | India | National Family Health Survey-4 & National Sample Survey | Not reported | Multistage stratified sampling | Women of reproductive age | 15-49 | hemoglobin< 12g/dL | 6,93,756 | 3,68,384 | 53.1 | 52.98-  53.22 | Daily dietary iron intake |
| Sohan Kumar Sharma et al | 2016 | Jan -Jul 2015 | Rajasthan | Observational study | Tertiary hospital | Purposive sampling | Males or females | ≥18 | 30 μg/L | 150 | 114 | 76 | 68.57-  82.13 | Anaemia |
| A S Ahankari et al | 2017 | Jan -Mar (2014) | Rural Maharashtra | Cross sectional survey | Villages | Multivariable sampling | Adolescent girls | 13-17 | Sahli's method (Hb level <11 g/dL) | 1010 | 878 | 86.93 | 84.71-  88.87 | Low fruit consumption, delayed menarche, high menstrual flow |
| S.V.Madhu et al | 2016 | Nov 2011-Apr 2013 | Delhi | Case control study | Not reported | Cluster sampling | Not reported | Not reported | ≥50.0 gm/l and <100.0 gm/l | 122 | 62 | 50.82 | 42.06-  59.53 | Diabetes mellitus |
| Rahul Jain et al | 2017 | Nov 2014-Apr 2016 | North India | Prospective interventional study | Pediatric department | Not reported | Children | 6-36 months | serum ferritin levels (cut off 30 μg/L) | 100 | 85 | 85 | 76.72-  90.69 | Breath holding spells |
| Salman Hussain et al | 2019 | April 2017 - May 2018 | New Delhi | Cross sectional study | Hospital | Purposive sampling | Aged people | >50 | Hb level ≤13 g/dL in male and ≤12 g/dL in female | 323 | 123 | 38 | 32-  43 | lower SES & education |
| Jithin Sam Varghese et al | 2019 | 2015-2016 | India | NFHS-4 | States and districts | Stratified sampling | Women of reproductive age | 15-49 | children (Hb <11 g/dL) & women( Hb <12 g/dL) | 800,809 | 438843 | 54.8 | 54.69-  54.91 | Stunting, underweight |
| S Parks et al | 2019 | Jan (2012)-Dec (2016) | Rural India and Maharashtra | Prospective cohort study | Not reported | Purposive sampling | Pregnant women | Not reported | Hb (3–11 g/dl) | 180053 | 138059 | 76.68 | 76.48-  76.87 | Maternal death, neonatal mortality, stillbirth |
| Deena Thomas et al | 2015 | Nov 2011- Apr 2013 | New Delhi | Cross sectional hospital based | Not reported | Not reported | Adolescents | 10-18 | (Hb <12g/dL in 10-18 y girls and Hb<13g/dL in 15-18 y boys) | 200 | 41 | 20.5 | 15.49-  26.63 | vegetarianism, low intake of iron folate and B12 |
| Farhad Ahamed et al | 2018 | Jan -Dec 2014 | Haryana | RCT | Community based | Random sampling | Pregnant women | 22-24 | hemoglobin level <11.0 g/dl | 400 | 367 | 91.75 | 88.64-  94.07 | Poor IFA supplementation |
| Anita Shet et al | 2015 | Feb 2011- Aug 2012 | Southern India | Prospective cohort study | Institution | Purposive sampling | Children | 2-12 | (children aged 6–59 months, (Hb) <11.0 g/dl; 5–11 years, Hb <11.5 g/dl; ≥12 years, Hb <12.0 g/dl) | 240 | 113 | 47.1 | 40.86-  53.39 | Iron deficiency, HIV |
| Ranadip Chowdhury et al | 2018 | Jan 2010 – Feb y 2012 | Delhi | RCT | Low -middle socioeconomic setting | Not reported | Children | 6-30 months | vitamin-D deficient (<10ng/ml) | 1000 | 331 | 34.5 | 30.25-  36.08 | vitamin D deficiency |
| Himanshu Arora et al | 2018 | Jul 2015-Jun 2016 | New Delhi | Observational study | Hospital | Purposive sampling | Adults | ≥18 | Hb <13 gm/dl (males) and < 12 gm/dl (females) | 275 | 211 | 76.73 | 71.39-  81.34 | Congestive heart failure |
| Alisha Chaubal et al | 2017 | Oct 2015 - 2016 | Western India | Observational study | Clinic patients | Not reported | Patients | Not reported | 13.1 g/dL | 64 | 34 | 53.1 | 41.08-  64.83 | Iron deficiency/ chronic fatigue |
| Krishnapillai Madhavan Nair et al | 2015 | Not reported | Telangana | RCT | Community | Stratified sampling | Boys and girls | 6-12 months 29-56 months | Hb <110 g/l and <120 g/l | 13,870 | 792 | 57.10 | 12.12-17.01 | Not reported |
| Caroline Katharina Stiller et al | 2020 | Dec 2014 - Apr 2015. | West Bengal | Not reported | Community | Cluster sampling | Children and mothers | 6-39 months | hemocue method | 595 | 558 | 93.78 | 91.54-  95.45 | Low income, poor |
| Rajvi Mehta et al | 2017 | Mar 2014 – Aug 2014 | Navi Mumbai | Cluster-randomized controlled trial |  | Cluster sampling | Women of reproductive age | 18-35 | Hb <12g/dL | 361 | 202 | 55.96 | 50.8-  60.99 | local diet consisting of non heme iron |
| Rahul Kaundal et al | 2019 | Jul 2017- Dec 2018 | North India | Cross sectional study | Not reported | Random sampling | Either gender | > 15 | y serum ferritin level < 20 ng/ml. | 62 | 62 | 100 | 94.17-  100 | Severe anemia |
| Karl Krupp et al | 2018 | Jan 2009 - 2012 | Karnataka | Prospective cohort study | Community | Purposive sampling | Pregnant women | 14-40 |  | 165400 | 1107 | 66.9 | 37-  97 | Lower education level of spouse, multi-parity, delivery type, and lack of involvement in household decision-making |
| Niraj S Ghatpande et al | 2019 | Not reported | Pune | Not reported | Not reported | Random sampling | Adolescent girls | 11-16 | (Hb <12 gm/dL)& (serum ferritin <15 mg/L) | 85 | 40 | 47.05 | 36.81-  57.57 | Lack of vitamin C–rich fruits and green leafy vegetable intake |
| Samuel P Scott et al | 2018 | Not reported | Maharashtra | Randomized controlled trial | School | Stratified sampling | Boys and girls | 12-16 | Anemic + ferritin<15 µg/L | 140 | 46 | 32.85 | 25.63-  41.01 | Cognitive performance |
| Lucas Gosdin et al | 2018 | Not reported | Bihar | Cross sectional study | Not reported | Multistage cluster probability | Children | 6-18 months | Hb 10.2 g/dL. | 5664 | 3897 | 68.8 | 67.58-  69.99 | Household wealth and social standing. |
| Kiran Bains et al | 2015 | Not reported | Punjab | Not reported | Community | Cluster sampling | Children | 6 months - 5 years | serum zinnc level< 60 µg/dL | 312 | 137 | 43.91 | 38.51-  49.46 | Vegetable, fruits and legumes |
| Lisa A Houghton | 2019 | Sept 2014 – Mar 2015 | New Delhi | Cross sectional study | Community | Random sampling | Young children | 12-23 months | Hb < 11.0 g/dL | 120 | 77 | 64.17 | 55.27-  72.19 | Iron deficiency |
| Oliver Didzun et al | 2019 | Jan 2015- Dec 2016 | India | Cross sectional study | Districts | Multistage sampling | Men & women | 15-54 | HemoCue | 739715 | 342677 | 46.3 | 22.7-  23.7 | Less household wealth, lower education, rural area, tobacco, and underweight |
| Kavitha C Menon et al | 2016 | Not reported | Maharashtra | Comparative study | Not reported | Stratified random sampling | Pregnant women | ≥24 | serum ferritin concentration 15 mg/L | 356 | 104 | 29.21 |  | Infant physical growth and behavior |
| Borde Deepak et al | 2015 | Jan 2009 – Nov 2014. | India | Retrospective observational study | Tertiary care hospital | Not reported |  |  |  | 335 | 174 | 51.94 | 46.6-  57.24 | Mortality |
| Vivek choudhury et al | 2015 | Not reported | New Delhi | Prospective cohort study | Clinic | Stratified random sampling | Infants | ≥34 weeks | serum ferritin ≤75 ng/mL | 90 | 23 | 25.5 | 16-  35 | Fetal brain development |
| N S Venkatesh Babu et al | 2017 | Not reported | Bangalore | Cross sectional study | Hospital | Not reported | Children | 3-12 | serum ferritin level 53.4% (±34.4) ng/dl | 120 | 38 | 31.6 | 23-  40 | Dental caries |
| Bhandar Gurbir Singh et al | 2019 | 2016-2018 | North India | Not reported | Tertiary care hospital | Not reported | Men & women | 18-81 | IDA: serum ferritin < 15 µg/L, soluble transferrin receptor protein > 1.76 mg/L | 94 | 30 | 31.91 | 23.36-  41.88 | Not reported |
| Siddharudha Shivalli et al | 2015 | May-Aug 2010 | Varanasi | Community based quasi experimental study | Community | Purposive sampling | Pregnant women | 13-28 weeks of gestation | Hb(<7g/dl) | 86 | 44 | 51.16 | 40.77-  61.45 | Increased demand, poor iron intake and improper dietary practices decreasing absorption of iron. |
| Aseem K Tiwari et al | 2018 | Oct-Dec (2015) | India | Randomized prospective study | Tertiary care hospital | Purposive sampling | Men & women | 18-51 | Hb level of ≥12.5gm/dl | 501 | 148 | 29.54 | 25.71-  33.68 | Not reported |
| Swarup Ghosh et al | 2018 | May 2016 – Mar 2017 | South Asia | Prospective observational study | Tertiary care hospital |  | Children | 6 months-12 years | plasma ferritin less than 50ng/mL | 42 | 36 | 85.71 | 72.15-  93.28 | Not reported |
| Suman Chakrabarti et al | 2018 | Not reported | North,  North-East,  South India | District Level Household and Facility Survey |  | A systematic, multistage stratified sampling design | Pregnant women | 15-49 | Hb cut-off of | 17138 | 11380 | 66.4 | 65.69-  67.1 | Sociodemographic & dietary intake |
| A D Jones et al | 2016 | Not reported | Andhra Pradesh | Cross sectional survey | Not reported | Random sampling | Adult men&women | ≥18 | Men(Hb 110-129 g/L) women (110-119 g/L) | 6217 | 1554 | 25 | 23.94-  26.09 | Cardiometabolic disease risk factors |
| Sriparna Bau et al | 2015 | Not reported | Varanasi | Prospective observational study |  | Not reported | Mother | 20-30 | Hb <11 g/dl and serum ferritin <12 ng/ml) | 142 | 71 | 49.95 | 0.39-  0.6 | Not reported |
| P Paul et al | 2019 | Not reported | Not reported | NFHS | Not reported | Multistage stratified sampling |  | Less than 5 & 15-24 |  | 80,539 | 39,569 | 49.13 | 48.78-  49.48 | Child marriage |
| P G Bansal et al | 2015 | Not reported | West Delhi | Community based randomized trial | Community | Random sampling | Adolescent girls | 11-18 | Hb 120 g/l | 794 | 446 | 56.17 | 52.7-  59.58 | Impaired cognitive performance, reduction in physical work capacity and productivity |
| Rakesh P S et al | 2015 | Not reported | Kerala | Cross sectional study | School | Stratified sampling | School children | 10-14 | HemoCue | 3200 | 1005 | 31.4 | 28.82-  33.04 | Irregular consumption of WIFS tablets |
| Ashutosh Singh et al | 2016 | Not reported | India | Not reported | Hospital | Stratified sampling | Patients | Not reported | Hb < 12.5 g/dl) | 400 | 100 | 25 | 20-  29 | Intake, impaired absorption, increase requirement and chronic blood loss |
| Nadia G Diamond Smith et al | 2016 | Feb -May 2009 | Chandigarh | Cross sectional study | Antenatal clinics | Random sampling | Pregnant women | 18-35 | hemoglobin less than 11g/dL | 120 | 87 | 72.5 | 63.91-  79.7 | Haemoglobin level |
| Tattari Shalini et al | 2019 | Oct 2014 – Sep 2016 | Hyderabad | Community based cross sectional study | Community based | Stratified sampling | Urban adults | 21-60 | ferritin concentrations<15 µg/L (ID) & ferritin concentrations<15 µg/L along with anemia (<13.0 g/dL in men, <12.0 g/dL in women) (IDA) | 300 | 209 | 69.7 | 20-  33.1 | Inadequacy of multiple micronutrients |
| Shashi Kant et al | 2018 | Jan 2015 – Dec 2016 | Haryana | Record based study | Hospital | Systematic sampling | Pregnant women | 24 | Hb below 11 g/dL | 8748 | 6827 | 78.04 | 77.2-  78.9 | Increased risk of maternal and fetal morbidity and mortality |
| Arun S Shet et al | 2015 | Nov 2014- Jul 2015 | Karnataka | Cluster-randomized controlled trial | Rural setting | Stratified sampling | Children | 12-59 | Hb ≥ 8 and ≤ 11 gm/dL | 270 | 60 | 22.3 | 17.67-  27.55 | Low dietary iron content of food |
| Jogmaya Pattnaik et al | 2020 | Mar 2015-Sep 2016 | Puducherry | Prospective study | Institution | Random sampling | Patients | <18 | ≥ 2 | 105 | 84 | 80 | 71-  87 | Cancer |
| Shivanand C Mastiholi et al | 2018 | Jan – Dec 2014 | Karnataka | Community based study | Primary health centres | Cluster sampling | Women | 15-39 | Sahli's method (Hb level <11 g/dL) | 770 | 724 | 94.03 | 92.13-  95.5 | SE and food insecure population. |
| Hagere Yilma et al | 2020 | Not reported | Odisha | Cluster-randomized controlled trial | Community | Cluster sampling | Women of reproductive age | 15-49 | HemoCue Method | 4000 | 2,040 | 51 | 49.45-  52.55 | Less education and Scheduled Tribes |
| Daniella Anne L Chyne et al | 2017 | Not reported | North east india | Community based cross sectional study |  | Random sampling | Children | 5 | less than 11 g/dl | 603 | 410 | 67.99 | 64.16-  71.59 | Undernutrition |
| Andezhath K. Susheela et al | 2016 | Not reported | East delhi | Institution based cross sectional study | School | Random sampling | Adolescent girls | 10-17 | Hemocue 201+ Angel Holm Sweden | 943 | 538 | 57.05 | 53.87-  60.17 | Higher fluoride |
| Rajesh Kashyap et al | 2014 | Not reported | Lucknow | Prospective study | Not reported | Not reported |  |  | (Hemoglobin < 6 g/dL | 66 | 17 | 25.75 | 16.75-  37.44 |  |
| Thulasingam Mahalakshmy et al | 2018 | Not reported | Puducherry | Mixed method study | Schools | Multivariable sampling | School adolescents | 14-19 | Not reported | 311 | 133 | 42.77 | 37.39-  48.32 | Poor awareness |
| Suja Karkada et al | 2018 | Not reported | Udupi | Quasi experimental study | Semi-rural setting | Cluster sampling | Adolescent girls | 13-14 | 11.03 g% ± 0.57 | 410 | 234 | 57.07 | 52.24-  61.77 | Nutritional education, dietary interventions |
| [Leila M. Larson et al](https://www.ncbi.nlm.nih.gov/pubmed/?term=Larson%20LM%5bAuthor%5d&cauthor=true&cauthor_uid=29947323) | 2018 | 2012 and 2014 | Rural Bihar | Cluster-randomized controlled trial |  | Cluster sampling | Children | 6 - 18 months |  | 8652 | 6229 | 72 |  |  |
| Sriparna basu .et al | 2016 |  | Varanasi | Prospective observational study | Neonatal Unit, Department of Pediatrics, Institute of Medical Sciences, BHU | Purposive sampling | Anemic mothers | 20-30 | hemoglobin < 70 g/L and serum ferritin < 12 μg/L | 45 | 30 | 66.67 | 52.07-  78.64 |  |
| Mallupudi et al | 2019 |  |  |  |  |  | Pregnant ladies and mothers |  |  | 48 | 28 | 58.33 | 44.28-  71.15 |  |
| Michael | 2017 | Jun 2009- Aug 2010 | West Bengal |  | RCT |  | Women of reproductive age | 18-55 |  | 126 | 83 | 65.87 | 57.23-  73.57 |  |
| Rekha et al | 2017 | Apr 2015-Oct 2015 | Patna, Bihar | Cross sectional study | Clinic | Stratified sampling | Adolescents | 10-19 |  | 200 | 100 | 50 | 43.14-  56.86 | Increased iron demand, menstrual blood loss, infection, worm infestation etc. |
| Rita Panyang et.al. | 2018 | 2015–2016 | Tea gardens of Dibrugarh, Assam | Cross-sectional study | Community-based | Random sampling | Adult females | 15-44 |  | 770 | 151 | 19.61 |  |  |
| Mullapudi Venkata Surekha et.al. | 2020 |  |  | Cross-sectional study | Institution based | Systematic random sampling | Pregnant women between 36 and 42 weeks of gestation | 18-45 |  | 200 | 118 | 19.61 | 16.96-  22.56 | Poor compliance to tablets supplied by the government associated vitamin B12 and folate deficiencies |
| Kashish Grover et.al. | 2020 | Jan 2018 to Dec 2018 | Pt. B.D. Sharma PGIMS, Rohtak | Cross-sectional study | Community-based | Universal sampling method | Pregnant women | Avg. Age = 26.55 ± 3.46 years |  | 408 | 348 | 85.29 | 81.52-  88.4 | The association of SE factors, dietary factors such as vegetarian diet, worm infestation, use of iron utensils, consumption of tea with meals and consumption of jaggery with anemia status |
| Giridhar Kanuri et.al. | 2016 | 2013–2014 | St. Johns Medical College and Hospital, Bangalore | Prospective, cross-sectional study | Tertiary medical center oncology clinic |  | Cancer patients |  | Access 2 Immuno analyzer | 218 | 139 | 63.76 | 57.19-  69.85 | High prevalence of ID in cancer patients |
| Aakriti Gupta et.al. | 2020 | 2015-2016 | District Nainital, Uttarakhand state, India | Cross-sectional study | Community-based | Cluster, home-to-home | Elder people | Mean age of males= 69.3 ± 7.4 years and females= 67.9 ± 7.2 years. |  | 958 | 881 | 91.96 | 90.07-  93.52 | Dietary intake and adequacy of nutrients was found to be significantly |
| Dheeraj Sharma et.al. | 2019 |  | PGIMER, Chandigarh | Prospective observational study | Hospital-based |  | Elder people | >=60 |  | 105 | 26 | 24.76 | 17.49-  33.81 | Multiple myeloma, back pain, renal failure, hypercalcemia |
| Dipshikha Maiti et.al. | 2019 | Apr 2016 and Sep 2016 | Teaching hospital in Kolkata | Prospective, cross-sectional study | Hospital-based |  | Children | 1–168 months |  | 697 | 296 | 42.32 | 38.7-  46.02 | Normocytic erythrocytes, folate deficiency |
| Julia L. Finkelstein et.al. | 2019 |  | Bangalore, Southern India | A prospective observational | Clinic-based |  | Pregnant women | At least 18 years of age, <=41 years | Biomarkers Reflecting Inflammation and Nutritional Determinants of Anemia (BRINDA) | 366 | 176 | 48.09 | 43.02-  53.2 |  |
| Sambedana Mohanty et.al. | 2020 | Oct 2014 Oct 2016. | Slums of Berhampur, Odisha | Cross-sectional study | Field-based |  | Adolescent girls |  |  | 160 | 144 | 90 | 84.37-  93.75 |  |
| PG Hariprasad et.al. | 2017 | July–Dec 2015 | SAT Hospital, Government Medical College, Thiruvananthapuram | Cross-sectional | Hospital-based |  | Children with diagnosed cases of cerebral palsy | 1-16 years | GMFM and FIM | 41 | 31 | 75.61 | 60.66-  86.18 | Poor dietary intake and feeding difficulties |
| J Clin Diagn Res et.al. | 2017 | 2015-16 | Government Medical College, Nalhar, Haryana | Case-control study | Institution based |  | Sputum positive PTB patients | 15-60 |  | 200 | 162 | 81 | 75-  85.83 | Tuberculosis cause anemia |
| Sankalp Dudeja et.al. | 2019 | 2 years | A children hospital in North India | Prospective observational study | Hospital-based |  | Children | 1–18 | Peripheral neuropathy and as per TNSr | 30 | 13 | 43.33 | 27.37-  60.8 |  |
| Arun Fotedar et.al. | 2018 | Nov 2015 to Feb 2016 | Three pediatric outpatient clinics New Delhi | Cross sectional study | Clinic-based |  | Children | 12-24 months |  | 66 | 28 | 42.42 | 31.24-  55.44 | Male gender and low birth weight |
| Manisha Nair et.al. | 2016 | Jan –Jun 2015 | Assam | Retrospective cohort study | 5 government medical colleges in Assam. |  | Pregnant women >20weeks of gestation |  | INTERGROWTH-21st too | 1007 | 651 | 64.65 | 61.65-  67.54 |  |
| Vijayram Reddy K et.al. | 2020 | Sep 2015 to Aug 2017 | (Kasturba Hospital, Manipal, Karnataka | Prospective study | Hospital-based |  | Whole-blood donors | 18-59 |  | 374 | 97 | 25.94 | 21.76-  30.61 |  |
| Matthew Little et.al. | 2018 |  | Rural Tamil Nadu, India | Cross-sectional study | Household based, 17 villages | Random sampling | Men and non-pregnant women from rural tamil nadu | >=20 | Food Frequency Questionnaire (FFQ), WHO guidelines | 753 | 370 | 49.14 | 45.58-  52.71 |  |
| Rambha Pathak et.al. | 2016 | Feb 2011 to Jan 2012 | Ambala school | Case-control study | Community-based | Multistage cluster sampling | Children | 6-12 | WHO/UNICEF guidelines | 540 | 380 | 70.37 | 66.39-  74.07 | Lower circulating T4 and T3 concentrations and higher thyrotropin concentrations |
| Ningappa Asha Rani et.al. | 2017 | Jun to Aug 2015 | Adichunchanagiri Institute of Medical Sciences | Cross-sectional study | Institution based | Convenient sampling | Healthy undergraduate medical students of both genders | Majority were from 17-20 |  | 289 | 45 | 15.57 | 11.84-  20.2 | Age, gender, physiological, nutritional and environmental factors and SES |
| Anirudh Mukherjee et.al | 2019 |  | Uttarakhand | Retrospective | Hospital-based |  | new cases of tuberculosis | >18 |  | 252 | 87 | 34.52 | 28.92-  40.58 |  |
| Nupura A. Vibhute et.al. | 2019 |  | Western Maharashtra | Cross-sectional study |  | Random sampling | Nonpregnant, unmarried female students | 15-49.9 |  | 300 | 86 | 28.67 | 23.85-  34.03 | Poor eating habits |
| Surekha Kishore et.al. | 2020 |  | Uttarakhand | Cross-sectional study |  |  | Male and female | < 5 , 5–14 and ≥15 |  | 5776 | 3072 | 53.19 | 51.9-  54.47 |  |
| P.M. Siva et.al. | 2016 | Apr 2013 to May 2014 | Ettumanoor panchayat, central Kerala, India. | Cross-sectional study | Field-based | Random sampling | Adolescent girls | 10-19 |  | 257 | 54 | 21.01 | 16.47-  26.4 | Presence of ova or cyst in stool |
| Shobha P Shah et.al. | 2016 | Apr -Jun 2013 | Tribal villages of Jhagadia | Cross-sectional study | Community-based |  | Adolescent girls and boys | 10-19 | HemoCue method | 244 | 174 | 71.31 | 65.34-  76.62 |  |
| Abhishek Pathania et.al. | 2019 | May 2015 to Oct 2015 | Old age homes, Delhi | Cross-sectional study | Cluster, old-age homes | Random sampling | Elderly people both men and women |  | HemoCue Hb 201+ system | 334 | 229 | 68.56 | 63.39-  73.3 | Gradual decrease in erythropoietin production by the kidneys |
| Sai Santhosh Vadakattu et.al. | 2019 | 2015 | Hyderabad | Cross-sectional study | Community-based |  | Urban elderly; both genders | >=60 |  | 282 | 58 | 20.57 | 16.26-  25.67 |  |
| Sathya P Manimunda et.al. | 2017 | 2012-13 | Aandn Islands | Cross-sectional study | Population-based | Multistage random sampling | Preschool children | 6-59 months | Household Food Insecurity Access Scale (HFIAS) | 1259 | 1019 | 80.94 | 78.68-  83.01 |  |
| Meenakshi Khapre et.al. | 2020 | 2018-19 | Rishikesh, India | Cross-sectional study | Community-based | Cluster, ward sampling | Adolescent girls | 10-19 |  | 400 | 317 | 79.25 | 75.01-  82.94 |  |
| Manju Mehrotra et.al. | 2018 | Feb - Jul 2017 | Port Blair, Andaman and Nicobar Islands | Cross-sectional study |  |  | Pregnant women | 12–40 |  | 786 | 400 | 50.89 | 47.4-  54.37 |  |
| Maninder Kaur et al | 2018 | Mar 2009 to Oct 2010 | North India- Punjab and Haryana | Cross-sectional study | Community-based | Random sampling | Post-menopausal women; women having at least 1 year natural amenorrhea | 45-80 |  | 250 | 213 | 85.2 | 80.27-  89.07 | Decline in hemoglobin levels |
| Ravishankar Suryanarayana et.al. | 2017 | Mar 2013-Jan 2015 | Kolar Taluk | Prospective, observational | Community-based study. | Multistage sampling technique | Pregnant women | 12–40 |  | 446 | 278 | 62.33 | 57.75-  66.7 | Maternal and fetal risks |
| I. K. Rohisha et al | 2019 | Not reported | Kerala | Cross sectional study | Tribal settlements | Cluster sampling | Women of reproductive age | 18-45 |  | 445 | 397 | 89.21 | 85.98-  91.76 | Not reported |
| [Shuchismita Behera et al](https://www.ncbi.nlm.nih.gov/pubmed/?term=Behera%20S%5bAuthor%5d&cauthor=true&cauthor_uid=27127647) | 2016 |  | Odisha | Cross-sectional community-based survey | Community | Random sampling | Children | <12 | Hb <11 g/dL | 313 | 194 | 61.98 | 56.49-  67.18 | Concomitant acute infection |
| Gustavo Corrêa et al | 2017 | Jan -Dec 2015 | Chhattisgarh, Andhra Pradesh and Telangana |  | Tribal settlements | Cluster sampling | Tribal pregnant women | 25-30 | RDT - HemoCue® Hb 301 | 563 | 520 | 92.36 | 89.87-  94.28 | Malaria |
| Susmita Sharma et al | 2019 | Jan 2018 - Jun 2019 | Haryana | Retrospective study | Tertiary care hospital | Random sampling | Female | 24 | e less than 11 g/deciliter (gm/dl) | 388 | 264 | 68.04 | 63.24-  72.48 | low SES poverty, poor access to health in rural areas, education |
| Anju Barki et al | 2016 | Not reported | Assam | Hospital |  | Cluster sampling | Both | 1-65 | HPLC | 1200 | 28 | 2.33 | 1.62-  3.35 | Carrier detection and awareness |
| Beena Koshy et al | 2020 | 2019 | Vellore | Longitudinal birth cohort study. |  | Urban slum, Vellore, India | Children | 2-5 | Blood Pb levels (BLL) | 251 | 228 | 90.84 | 86.63-  93.82 |  |
| Baraturam Bhagrati Bhaisara et al | 2019 | 2009 | Mumbai, Maharashtra, India | Cohort study | Department of Pediatrics, HBT Medical College & Dr R N Cooper Municipal General Hospital | Selective | HIV - infected pediatric patients | 18–24 months | < 11.2 g/dL | 489 | 86 | 17.59 | 14.47-  21.21 | - |
| Archana D Rathod et al | 2016 | 2013 | Yavatmal,Maharashtra | Cross- sectional study | Gynecological OPDdepartment | Selective | All adolescent girls with puberty menorrhagia | 10-19 | < 9 g% | 655 | 17 | 2.6 | 1.63-  4.12 | Severe anemia resulting from puberty menorrhagia |
| Tejasav Sehrawat et al | 2018 | 2013-2015 | Chandigarh | Cross- sectional study | IPD and OPD, Government Medical College and Hospital | Selective | Liver cirrhosis patients | Random | - | 100 | 90 | 90 | 82.56-  94.48 | - |
| Navneet Khandelwa et al | 2020 | 2015-2016 | Ujjain, MP | Cross- sectional study | NRU | Selective | Children | < 13 | Hb<7 gm% | 100 | 67 | 67 | 57.31-  75.44 | - |

| **Table 4: Characteristics of the studies identified in the systematic review of the prevalence of Folic acid deficiency among people in India.** | | | | | | | | | | | | | | |
| --- | --- | --- | --- | --- | --- | --- | --- | --- | --- | --- | --- | --- | --- | --- |
| **Author** | **Year of publication** | **Year of study** | **Place of study** | **Study design** | **Study setting** | **Sampling technique** | **Study population** | **Age group of SP (Yrs)** | **Scales used** | **Sample size** | **Micronutrient deficient samples** | **Prevalence** | **95 CI%** | **Determinants/Risk factors** |
| Prajkta Bhide et al | 2018 | 2017 | Pune | Observational study | Government  hospital , Pune | Simple  random sampling | Pregnant women | Pregnant women of  11+/-3 week of gestation | ID vit FA microbiological essay kit | 584 | 142 | 24.32 | 21.02-27.96 | No proper educational knowledge. |
| Akriti Gupta et al | 2017 | 2015 | 3 districts of Himachal Pradesh | Community based cross   sectional | Government schools of the districts | Cluster sampling | Children | 6-18 | Chemilumin escence immunoassay method,less than 4 ng/ml considered as deficient | 215 | 3 | 1.40 | 0.48-4.03 | Low intake of pulses and green leafy vegetables. |
| Deena Thomas et al | 2015 | Nov 2011-Apr 2013 | New Delhi | Cross-sectional observational study | Lady Hardinge Medical College | Simple random sampling | Anemic adolescents | 10-18 | Automated immunoassay system using Beckman Coulter Access-2 | 200 | 159 | 79.50 | 73.37-84.51 | Low dietary intake |
| Prava M R Adhikari et al | 2016 | 2015 | South India | Cross-sectional observational study | Hospital, tertiary care South India | Stratified random sampling | HIV positive patient | All ages | Carbonyl metallo-immunoassay method for folate 2.7-34.0 ng/ml | 200 | 60 | 30 | 24.07-39.68 | Less micronutrient supplements |
| Sanket K et al Mahajan et al. | 2015 | 2014 | Maharashtra | Cross-sectional observation study | Medicine department, Institute of Krishna Institute of Medical Sciences University, Karad | Purposive sampling | Patient with symptom | Not mentioned | ACS: 180 folate assay using direct chemiluminescent technology | 100 | 26 | 26 | 18.40-35.37 | Nutritional deficiency, food-cobalamin malabsorption, pernicious anemia, dietary folate depletion. |
| [Sankalp Dudeja et al.](https://pubmed.ncbi.nlm.nih.gov/?term=Dudeja+S&cauthor_id=31514565) | 2019 | Not mentioned | North India | Prospective observational study | A children hospital in North India | Simple random sampling | Children with acute lymphoblastic anemia | 1-18 | Automated chemiluminescent immunoassay (Beckman Coulter Access-2). Deficiency was defined as levels below 5 ng/ml | 30 | 6 | 20 | 9.51-37.31 | Inadequate dietary intake. |
| Nirmalya Roy Moulik et al | 2017 | From December 2011 through November 2013 | Uttar Pradesh, Lucknow | Cross sectional study | A tertiary care teaching hospital | Simple random sampling | Children undergoing maintenance chemotherapy for acute lymphoblastic leukemia | 7.0 ± 3.3 | Electrochemiluminescence (using Cobas e 411 analyzer) prior to each cycle of maintenance folate deficiency was defined as serum folate level less than 4 ng/ml.10 | 52 | 29 | 55.77 | 42.34-68.41 | Low socio-economic status as well as vegetarians. |
| Prabha M Adhikari et al | 2016 | Not mentioned | South India | Cohort study | Tertiary care hospital attached to a medical college | Stratified random sampling | HIV positive patient | 38.00 ± 8.12. | Carbonyl metallo immunoassay method and reference range that was taken for this study was 2.7–34.0 ng/ml | 150 | 41 | 27.33 | 20.83-34.96 | Cachexia induced malnutrition and decreased absorption of folic acid due to advanced disease |
| M. Sivaprasad et al | 2016 | October 2012 to September2014 | In Hyderabad city and Khammam town of the Telangana state. | Community-based cross-sectional study | South Indian  urban setup | Stratified random  sampling | Urban adult population | 21 to >60 | Radio immunoassay (RIA) method using a dual-count solid-phase noboil RIA kit (Siemens Medical Solutions Diagnostics, Los Angeles, Calif., USA) , <3 ng/ml were considered deficient | 630 | 75 | 11.90 | 9.60-14.66 | Mixed diet, elderly aged, less vegetable consumption. |
| Himanshu Arora et al | 2018 | 1st July 2015 to 30th June 2016. | New Delhi | Cross sectional study | Hospital (Dharma Vira Heart Centre, Sir Ganga Ram Hospital) | Simple random sampling | Patients having clinical criteria for heart failure | 18 +, mean age - 62.72 | < 4 ng/ml – folate deficiency | 275 | 44 | 16 | 12.14-20.80 | Dietary intake is inadequate, during times of digestive blood loss or menstrual period and states that excessively |
| [Samiksha Singh](https://www.ncbi.nlm.nih.gov/pubmed/?term=Singh%20S%5BAuthor%5D&cauthor=true&cauthor_uid=32153830) et al | 2017 | December 2012 to November 2013 | NIN labs in Hyderabad (100 km) | Cross sectional study | Rural areas of Mahbubnagar district, Telangana India | Multi-stage stratified random sampling method | Married or unmarried women | 15-35 | <3 ng/ml- Folate deficiency | 979 | 556 | 56.97 | 53.84-60.04 | Old age, child bearing age and breast feeding in women |
| Manish K. Yadav et al | 2016 | 2012-2013 | South India | Comparative study | Hospital | Simple random sampling | People suffering from megaloblastic anemia and suspect of being suffered with it as control | Average 40 | Competitive immuno assay method | 100 | 50 | 50 | 40.38-59.62 | Lack of insufficient amount of vitamin B9 in diet |
| Neha Berry et al | 2018 | January 2012 and December 2013 | World Medical Assembly Declaration of Helsinki | Prospective observational study | Tertiary  Referral  Hospital | Simple random sampling | Consecutive patients with documented CD | > 12 | 18.89 ± 12.42 ng/ml | 103 | 4 | 3.88 | 1.52-9.56 | Inadequate dietary intake |
| [Rati Jani et al](https://pubmed.ncbi.nlm.nih.gov/?term=Jani+R&cauthor_id=25898712) | 2015 | Mar-2012 | Inter Systems biomedical Ethics Committee, Mumbai | Cross sectional study | Sangamner Taluka, Ahmednagar District of Maharashtra | Multi-stage stratified random sampling method | Tribal Indian adolescents’ boys and girls | 14.0 ± 1.3 | Recommendations as RBC folate deficiency levels < 340 nmol/L | 224 | 112 | 50 | 43.51-56.49 | Poor consumption of green leafy vegetables(100g/day) and fruits (100g/day) |
| Jogamaya Pattnaik et al | 2019 | March 2015 to September 2016 | Puducherry | Cross-sectional study | JIPMER Puducherry, India | Stratified random sapling | ALL adults and pediatric patients | 0.6-55 | Grade ≥ 2 anemia and patient with serum level was 0.35-5.33 | 84 | 38 | 45.24 | 35.04-55.56 | Malnutrition |
| Lisa A. Houghton et al | 2019 | Sep 2014 - Mar 2015 | New Delhi | Cross-sectional study | Organized health camp | Convenience sample | Young children | 12 to 23 months | ELISA | 120 | 45 | 37.50 | 29.35-46.42 | Malnutrition |
| Bridget Ann Knight et al | 2015 | 2014 | South India | Cross sectional study | University Hospital Birmingham NHS Trust, UK | Systematic sampling | Pregnant women | 28 weeks | Microbiological assay assay | 995 | 299 | 30.05 | 27.28-32.97 | Inadequate of dietary intake |
| Aakriti Gupta et al | 2017 | 2015–2016 | Nainital, Uttarakhand | Community-based cross-sectional study | Nationwide survey conducted by national nutritional monitoring | Cluster sampling | Aged person | 69.8+/-7.6 | Dietary parameter, | 225 | 183 | 81.33 | 75.73-85.88 | Lack of dietary intake of essential micronutrients |
| Tattari shalini et al. | 2018 | October 2014 -September 2016 | Hyderabad city, Telangana state | Community based cross sectional study | Health camp | Simple random sampling | All except pregnant and lactating women. | 21-85 | Hemoglobin level<13.0 g/dl in men, Hb <12.0 g/dl | 300 | 161 | 32.20 | 28.25-36.42 | No proper dietary intake and individual micronutrient adequacy |
| G krishna Swetha et al | 2015 | July 2009 and February 2011 | Hyderabad, India. | Observational | Two orphanages | Simple random sampling | HIV-positive children aged | Between 1 1/2 and 15 | Siemen’s vitamin B12 and folate dual count kit (Healthcare, USA). Not mentioned | 77 | 37 | 48.05 | 37.25-59.03 | Inadequate dietary intake |
| Nand Kishor Shinde et al | 2016 | December 2012 to August 2014 | Northern India | Observational cross section | Hospital | Stratified random sampling | Patients of CPC who had undergone definitive surgery and closure of a protective stoma | 14 | Not mentioned | 31 | 7 | 22.58 | 11.39-39.81 | Lack of micronutrient supplements in diet |

| **Table 5: Characteristics of the studies identified in the systematic review of the prevalence of Vit A deficiency among people in India.** | | | | | | | | | | | | | | |
| --- | --- | --- | --- | --- | --- | --- | --- | --- | --- | --- | --- | --- | --- | --- |
| **Author** | **Year of publication** | **Year of study** | **Place of study** | **Study design** | **Study setting** | **Sampling technique** | **Study population** | **Age group of SP (Yrs)** | **Scales used** | **Sample size** | **Micronutrient deficient samples** | **Prevalence** | **95 CI%** | **Determinants/Risk factors** |
| Ashok kumar Jindal et al | 2018 | 2018 | Dholpur, Rajasthan | Cross- sectional study | Swatantra Sainanilate Dr.Mangal Singh district hospital | Selective | Random; no exclusion | 13-55 | 0.25-0.3 mg/l,  0.20-0.24 mg/l,  <0.20mg/l | 54 | 54 | 100% | 96.3-100 | Children are at higher risk of vitamin a deficiency owing to their greater requirement for growth. |
| Pathak S et al | 2017 | April 2015- Sept 2016 | Waghodia, Vadodara | Cross sectional and observational study | Pediatric ward and NRC (Nutritional Rehabilitation Centre) of Dhiraj Hospital | Selective | Children between 6 months to 5 years. | 6 months to 5 years | <0.3 mg/L | 200 | 12 | 6% | 3.47-10.19 | Reported Limitation of study- didn't find reason for VAD |
| G. D. Bhambhani et al | 2015 | Sept 2014- aug 2015 | Bhavnagar district, gujarat | Cross- sectional study | School | Random | Adolescents | 10 - 19 | <0.3 mg/l | 867 | 53 | 6.11% | 4.7-7.91 | Reported limitation of study- didn't find reason for VAD |
| Ali Jaffar Abedi et al | 2015 | 2015 | Lucknow, Uttar Pradesh | Cross- sectional study | Rural and urban Lucknow | Random | Pre-school children | 12months - 59 months | <0.4 mg/l | 400 | 25 | 6.25 | 4.27-9.06 | Village/Mohalla, religion, social class, education of head of the family, age group and sex of the child, height/age z score, weight/age z score |
| Divya Elizabeth Muliyil et.al. | 2019 | Dec 2015- Feb 2016 | Jawadhi hills, Tamil Nadu | Cross-sectional study | Residents of Jawadhi hils- Children and women of childbearing age | Simple random sampling | Interviewer -administered structured questionnaire | 1-8 (C), 15-45 (W) | WHO classification of BMI for Asians | 377 | 25 | 6.63% | 4.53- 9.6 | Level of education of the head of the household and pregnancy |
| Shivali Suri et.al. | 2017 | 2011 | RS Pura block of Jammu district | Cluster study | Community based | Multistage random sampling | Children | 12-60 months | Helen Keller International Food-Frequency Questionnaire (HKI-FFQ), Uday Pareek scale | 750 | 213 | 28.4% | 25.29- 31.73 | Low bioavailability from plant sources of Vitamin A |
| Lapam Panda et.al. | 2019 | Aug 2016- July 2017 | Native tribal district, Ravaged, Odisha | Cross-sectional comparative study | School and hospital | Systematic random sampling | Tribal school students | 5-16 | Declaration of Helinski for research involving human beings | 153107 | 70 | 0.05% | 0.04- 0.06 | VAD was only found in children of native schools and not in those of urban schools: general health awareness, ecosystem of area, proximity of care |
| Thingnganing Longvah et. Al. | 2018 | 2012-2013 | Chakhesang tribe, North-East India | Cross sectional descriptive study | Community based | Systematic random sampling | Children, adult women, adult men | <5 | Middle-upper arm circumference (MUAC). Food insecurity experience scale (FIES) | 811 | 227 | 27.99 | 25.01- 31.18 | Low serum retinol levels, young children are unlikely to consume sufficient dietary sources of beta-carotene, low intake of oils-hampered absorption and bioavailability of fat-soluble vitamins |
| Lisa A. Houghton et.al. | 2018 | Sep 2014- March 2015 | Slum area Badarpur, New Delhi | Cross-sectional study | Household-based | Simple random sampling | Children | 12-23 months | Standardized micronutrient and ln (CRP) values | 120 | 20 | 16.67 | 11.06- 24.35 | The 17% VAD was low |
| Veer Singh et.al. | 2017 | June 2012- Aug 2014 | Urban and rural schools of West Uttar Pradesh | Cross-sectional study | School-based | Systematic random sampling | School students | 5-15 | Snellen's VA chart at 6m | 4838 | 101 | 2.09% | 1.72- 2.53 | More in rural population |
| Anita Shet et.al. | 2015 | Feb 2011- Aug 2012 | St. John's Hospital, Bangalore, NIRT, Chennai, YRG Centre for AIDS Research and Education | Prospective cohort study | Institute based | Systematic random sampling | Prenatally HIV infected children | 2-12 | WHO criteria for anemia | 240 | 43 | 17.92 | 13.59- 23.27 | Children with HIV having anemia |
| Daniella Anne L. Chyne et.al. | 2018 | 2016 | Khasis of North-east India | Cross-sectional study | Community based | Random sampling | Children and mothers | < 5, | Food insecurity experience scale | 1103 | 328 | 29.74 | 27.12- 32.5 | Low intake of green leafy vegetables and meat, low purchasing capacity and lack of awareness |
| Deepanshu Agrawal et.al. | 2020 | Dec 2017- Sep 2018 | Raipur, Chhattisgarh | Prospective, cross-sectional study | School- based | Random sampling | School children | 5-15 | WHO grading system for Bitot's spot or Keratomalacia | 1557 | 156 | 10.02 | 8.63- 11.61 | Reported Limitation of study- didn't find reason for VAD |
| PG Hariprasad et.al. | 2017 | July-Dec 2015 | SAT hospital, government Medical College, Thiruvananthapuram | Cross-sectional study | Hospital based, Tertiary care Centre | Simple random sampling | Children | 1-16 | Gross Motor Function Measure (GMFM), functional ability by a modified functional-independent measure (FIM) | 41 | 31 | 75.61 | 60.66-86.18 | Poor dietary intake and feeding difficulties |
| Lapam Panda et.al. | 2020 | 2018-2019 | Tribal district of Odisha | Cross-sectional, observational study | Tribe based | Systematic random sampling | School children with ocular signs/symptoms of VAD | Age range: 5-6 | Declaration of Helinski for research involving human beings | 4801 | 207 | 4.31 | 3.77-4.92 | Didn't identify risk factors leading to VAD |
| G. Krishna Swetha et.al. | 2015 | July 2009-feb 2011 | Two orphanages at Hyderabad | Observational, longitudinal study | Orphanage based | Simple random sampling | HIV positive children | 1.5-15 | Seca weighing scale | 77 | 11 | 14.29 | 8.17-23.8 | HIV |

| **Table 6: Characteristics of the studies identified in the systematic review of the prevalence of Vit D deficiency among people in India.** | | | | | | | | | | | | | | |
| --- | --- | --- | --- | --- | --- | --- | --- | --- | --- | --- | --- | --- | --- | --- |
| **Author** | **Year of publication** | **Year of study** | **Place of study** | **Study design** | **Study setting** | **Sampling technique** | **Study population** | **Age group of SP (Yrs)** | **Scales used** | **Sample size** | **Micronutrient deficient samples** | **Prevalence** | **95 CI%** | **Determinants/Risk factors** |
| Krishna DeeptiMogili, et al | 2018 | March 2016 to March 2017 | South India | Cross-sectional study | Infertility centre, South India | Random sampling | Infertile PCOS women | 20 - 40 | Menstrual cycles, waist circumference, fasting blood sugar, fasting lipid profile, thyroid stimulating hormone levels and serum vitamin D levels, diabetes, hypertension, obesity, and ischemic heart disease | 256 | 20.3 % (52/256) | 70.30% | 64.45-  75.57 | Age, BMI, waist circumference, mean systolic and diastolic pressure, prior history of diabetes mellitus, & hypertensive disorder, mean fasting and postprandial blood sugar levels, TSH, total cholesterol and triglyceride levels. |
| [WarishaMariam, et al](https://pubmed.ncbi.nlm.nih.gov/?term=Mariam%20W&cauthor_id=31336515) | 2019 | January 2017 to December 2017 | Delhi, India. | Cross-sectional study | Tertiary care hospital in Delhi, India. | Random sampling | Adult women with T2DM on treatment for at least 6 months | Adult women | Women with Type 2 Diabetes Mellitus | 100 | 77% | 77% | 67.85-  84.16 | Diabetes, lower self-reported sun-exposure, non-vegetarian, high TAG levels had more Vitamin D deficiency. |
| PallaSuryanarayana, et al | 2018 | 2014- 15 | Hyderabad, South India | Cross-sectional study | Urban elderly population in Hyderabad | Random sampling | Males and Females of ≥ 60 years | ≥ 60 | Blood pressure, history of diabetes, history of dyslipidemia, socio-demographic particulars, lifestyles, physical activity, type of diet and history of non-communicable diseases (NCDS). | 298 | (57.2%) among men and (54.5%) among women. | 56.30% | 50.36-  61.56 | Increasing age, lack of exposure to sunlight, low dietary intake, an association of chronic diseases |
| Rose Mary J. Vatake ncherry et al. | 2019 | 3 months | South India | Cross sectional study | Tertiary care hospital | Not given | Hypertension people | 20 - 60 | <20 ng/ml, ARCHITECT 25-OH assay | 520 | 410 | 78.80% | 75.13-  82.14 | Not given |
| Shailender Prasad et al. | 2015 | June 2013 to May 2014 | New Delhi | Prospective Observational Study | Pediatric intensive care unit | Random | Critically ill children | 2mo - 12 years | <20ng/ml | 80 | 67 | 83.80% | 74.61-  90.25 | Age, Gender, Weight, Height and BMI. |
| UmeshKapil et al. | 2018 | 2015 to 2016 | Himachal Pradesh (Kullu and Kangra) | Community Based Cross Sectional Study | Schools above 1000mts. | Population proportionates to size | Healthy Children | 6 - 18 y | <20ng/ml | 1222 (Kangra: 610; Kullu: 612) | 495(Kangra) ;493(Kullu) | 80%(Kullu) 81%(kangra) | 78.55-  82.96 | Height, Weight and BMI. |
| NidhiChauhan et al. | 2019 | January 1, 2015 to October 31, 2015 | North India | Retrospective chart review | Child and adolescent psychiatric (CAP) clinic | Random | Psychiatric Illness | 4 - 15 | <20ng/ml | 40 | 32 | 80% | 65.24-  89.5 | Gender, age, education, religion, locality, duration of illness and multiple diagnoses. |
| Mini Sreedharan et al | 2018 | June 2012 to May 2013 | Kerala | Cross sectional study | Tertiary-care Pediatric Neurology centre, and day-care centre and school | Random | CBZ or VPA monotherapy treated patients | 2 -13 | <12 ng/ml, ELISA 96T kit | 56 | 9 | 16.10% | 8.69-  27.8 | Age, weight, height, BMI, average duration of exposure to sunlight per day, type of epilepsy, drugs used for treatment of epilepsy, duration of epilepsy, frequency of seizures per month, type of epilepsy, and duration of antiepileptic therapy |
| SangitaNangia Ajman et al | 2015 | January to December 2014 | Delhi | Not given | Kasturba hospital | Random | Pregnant burka clad women | 18 - 40 | <20ng/ml | 200 | 75 | 37.50% | 31.09-  44.39 | Preeclampsia, gestational diabetes mellitus (GDM), bony abnormality and mode of delivery |
| PradipMukhopadhyay et al | 2019 | Not given | West Bengal | Population based observational study | Under developed rural area | Random | Cardiometabolic concern or skeletal biochemical abnormality | 18 - 68 | <20ng/ml | 405 | 228 | 56.29% | 51.43-  61.05 | Height, weight, waist circumference, blood pressure and BMI. |
| SoumiSrimani et al | 2017 | 27th March, 2014 to 1st August, 2016 | West Bengal | Cross sectional study | Rural | Random | Post-menopausal women | 45 - 70 | <20ng/ml | 222 | 113 | 51% | 44.36-  57.4 | , Blood pressure (BP), waist circumference (WC), fasting blood glucose (FBG), triglycerides (TG) and high-density lipoprotein cholesterol (HDL-C) |
| C.P. Pal et al | 2016 | 1 November 2011 to 31 October 2013 | Agra | Not given | F Sarojini Naidu Medical Colleg | Random | Orthopaedic patients | 20 - 80 | <30ng/ml | 1132 | 1034 | 91.30% | 89.56-  92.84 | Age, Height, weight and BMI. |
| SaloniArora et al | 2017 | 2017 | Northern India | Cross-sectional study | Tertiary Care Center | Random sampling | Pregnant women with singleton pregnancy. | Adult women (age not given) | Demographic details, dietary history, past medical history, previous obstetric history, antenatal history, vitamin D tablets during pregnancy, its dose, and duration. | 200 | 86% | 86% had vitamin D deficiency, and 9.5% had vitamin D insufficiency | 80.51-  90.13 | Preeclampsia, increased Cesarean rate, and low birth weight babies |
| JhumaSankar et al | 2016 | July–Dec 2013 | New Delhi, India | Observational study | Pediatric intensive care unit (PICU), tertiary care centre. | Random sampling | Critically ill children | 1 month–17 years | Children on vitamin D supplementation, had received large doses for rickets or documented vitamin D deficiency in the past 1 year or steroids for at least 10 days before admission, or had recent kidney stones or chronic kidney disease. | 196 | 70% | 80% | 73.4-  84.64 | Association between length of ICU stay and vitamin D deficiency. |
| ChayanikaDutta et al | 2019 | July 2014 and June 2015 | Assam, India. | Cross-sectional study | Department of General Medicine, Assam Medical College & Hospital, Dibrugarh, Assam, India | Random sampling | Systemic lupus erythematosus patient | Not given | Systemic lupus erythematosus (SLE), without history of autoimmune disorders, hepatic or renal disorder, | 109 | 55.96% | 55.00% | 45.7-  64.06 | Age, years, High SLE disease activity (SLEDAI score > 10), Nephritis, Newly diagnosed SLE, Disease duration, months, Photosensitivity, Hydroxychloroquine treatment, Steroid treatment , Serum anti-sdna levels, IU/ml. |
| Rajesh Rajput et al | 2019 | Not given | Rohtak, Haryana, India | Cross-sectional study | Department of Obstetrics and Gynecology, Pt. B D Sharma Postgraduate Institute of Medical Sciences | Random sampling | Gestational diabetes mellitus (GDM) patients. | 50 | Demographic characteristics, socio-economic status, parity, family history of diabetes and/or hypertension and past history of GDM, history of smoking, alcohol consumption. | 50 | 44% of GDM women (22 out of 50) and 20% of normoglycemic women (10 out of 50). | 64.00% | 50.14-  75.86 | GDM, insulin sensitivity |
| K. D. Modi et al. | 2015 | June 2014 and December 2014 | India | Cross sectional, observational study | Two tertiary level hospitals | Random | Prediabetic and Type 2 diabetic patients | 18 - 65 | <20ng/ml | 606 | 516 | 85% | 82.1-  87.76 | Age, weight height, BMI, sex, family history of diabetes and duration of diabetes |
| Indar Kumar Sharawat et al. | 2019 | May to July 2011 | North western state of India | Cross-sectional study | Government Primary School | Random | Healthy school going children | 60 -120 months | 50 nmol/L, immunoradiometric assay | 100 | 65 | 65% | 55.24-  73.64 | Height, Weight, BMI and Age. |
| Ravikant Kumar et al. | 2017 | November 2013 and April 2015 | North India | Case control Study | M L N Medical College, and swarooprani Nehru (SRN) Hospital | Random | Patients with cirrhosis of the liver | 47.98 ± 12.13 | <20ng/ml, chemiluminescence immunoassay | 160 | 83 | 51.85% | 43.31-  58.48 | Height, Weight, Age and gender |
| UmeshKapil et al. | 2017 | 2014 to 2015 | Shimla district, Himachal Pradesh | Community-based cross-sectional study | Children residing at higher altitudes | Population proportionates to size | School students | 6 - 18 | <20ng/ml, chemiluminescence immunoassay | 626 | 582 | 93% | 90.69-  94.72 | Height, Weight and BMI. |
| Mamatha B Patil et al. | 2018 | Not given | Bangalore | Not given | Rajeswari Medical College and Hospital | Random | Female Type 2 diabetic patients | 30 - 60 | <20ng/dl, ARCHITECT 25-OH Assay kit | 156 | 144 | 92.50% | 87.04-  95.55 | Height, Weight, waist-hip ratio and BMI |
| Raman K. Marwaha | 2019 | Not given | Delhi, India | Cross-sectional study | Four fee paying schools in Delhi (Latitude N 28.38°, E 77.12°), India, | Random sampling | School children | Not given | Anthropometry measurements such as height, weight, and body mass index (BMI) | 615 | 51.50% | 100% | 46.98-  56 | Vitamin D supplementation |
| [RituKaroli et al](https://pubmed.ncbi.nlm.nih.gov/?term=Karoli+R&cauthor_id=26710398) | 2015 | Not given | Lucknow, India | Cross-sectional study | Divine Heart multispeciality hospital, Lucknow. | Random sampling | Group 1 comprised of patients with pulmonary tuberculosis, group 2 comprised of hospitalized patients who had non-tuberculosis medical illnesses and group 3 comprised of age and sex matched healthy controls | 18–60 | Age, sex, socio-economic status, demographic data and full medical history, including personal history of and/or contact with TB, drug and alcohol history. | Group 1 (n=100), group 2 (n=96) and group 3 (n=100) | Group 1: - 56%, group 2: - 35%, group 3:- 31% | 41% | 35.3-  46.42 | Pulmonary tuberculosis, Non-tuberculosis medical illnesses |
| [Pradeep Kumar et al](https://pubmed.ncbi.nlm.nih.gov/?term=Kumar+P&cauthor_id=26121736) | 2015 |  | Karanataka, India | Cross-sectional study | Department of Pediatrics, Cloudnine hospital, OAR Branch, Kodihalli, Murugeshpalya, Bangalore | Random sampling | Women in Labor and Cord Blood of Newborns |  |  | 106 women and their newborns | (70.7% mothers and 88 (83%) newborns |  | 70.74-  82.03 | Not given |
| [Suresh Kumar Angurana et al](https://pubmed.ncbi.nlm.nih.gov/?term=Angurana+SK&cauthor_id=28974139) | C | January to july 2017 | Chandigarh, india | Cross-sectional study | Pediatric intensive care unit, department of pediatrics, government medical college and hospital (GMCH), Chandigarh, India | Random sampling | Children with septic shock | Not given | Children with septic shock | 43 children | 69% | 72% | 54.9-  81.4 | Preexisting or acute liver disease, recent kidney stones, or chronic kidney disease, genetic/neurometabolic disorders, autoimmune, immunodeficiency disorders, and nephrotic syndrome. |
| RiteshAgarwal et al | 2018 | 1st January 2015 and 31st December 2016 | Chandigarh, India | Cross-sectional study | Postgraduate Institute of Medical Education and Research (PGIMER), Chandigarh, India | Random sampling | Patients with allergic bronchopulmonary aspergillosis (ABPA) and asthma. | Not given | Asthma, seruma .fumigatus-specific Ige>0.35 kilounit , presence of precipitating antibodies against A.fumigatus in serum, total eosinophil count >500 cells/µl and, (c) radiographic pulmonary opacities consistent with ABPA. | 188 and 108 subjects with ABPA and asthma and 50 healthy controls. | 70%, 64% and 65% of the healthy controls, asthmatics and ABPA subjects | 70% | 64.97-  74.59 | Asthmatic patients with or without ABPA. |
| Ranadeep Ray | 2019 | May 2016 till April 2017 | Delhi, India | Cross-sectional study | Department of Pediatrics, UCMS and GTB Hospital, Dilshad Garden, Delhi 110 095 | Random sampling | Healthy infants | 9-10 months | Birthweight more than 2.5 kg and were born at term gestation, were predominantly breastfed till 6 months of age, | 75 infants | Summer and winter: - 15.3%, 12.5% | 25.50% | 17-  36.39 | Childhood diseases, summer and winter. |
| TauseefAkhtar et al | 2019 | Not given | New Delhi, India | Cross-sectional study | Department of Medicine, Lady Hardinge Medical College and Dr. Ram manoharlohia Hospital, New Delhi, India. | Random sampling | Patients with Coronary Artery Disease and Association with Sun Exposure | 40 - 70 | Patients with Coronary artery disease, serum vitamin D levels along with serum and urine chemistries | 121 | 51.20% | 31% | 23.45-  39.72 | Serum calcium levels, serum albumin levels and sun exposure/day. |
| Kala Ebenezer et al | 2015 | January 18 through February 8, 2013 | Vellore, India | Cross-sectional study | Pediatric Intensive Care Unit of Christian Medical College, Vellore, India | Random sampling | Critically ill children | Not given | Data regarding the demographic profile, provisional diagnosis at the time of admission, past medical history, intake of vitamin D and sun exposure, presence of an underlying chronic illness and medications | 54 | 40.30% | 63.30% | 49.96-  74.87 | Chronic disease condition involving major organ systems present in 16 (30.8 %) children were: oncologic (6), cardiac (3), rheumatologic (2), neurologic (1) endocrine (1), hepatic (1), immunodeficiency (1), and renal (1). Shock (30.8 %), CNS conditions (23.1 %) and respiratory illnesses (21.2 %). |
| KirtikarShukla et al | 2016 | July 2011 June 2014 | Gurgaon, Haryana India | Retrospective cross-sectional study | Department of Clinical Biochemistry-Lab Medicine, Medanta The Medicity, Gurgaon, Haryana India | Random sampling | Healthy individuals | Not given | Vitamin D level, age and sex | 26,346 | 67 % male and 33 % female | 93 % | 92.69-  93.3 | Vitamin D level, age and sex |
| NighatYaseenSofi et al | 2017 | 6 months | Delhi, India | Cross-sectional study | OPD services of a tertiary care hospital. | Random sampling | Healthy women (controls) in the reproductive age group of 20–49 years. | 20–49 | Nonpregnant, nonlactating women of reproductive age without any history of chronic disease, Vitamin D intake or any other dietary supplementation, socio-demographic profile such as name, age, educational qualification, present occupation, and monthly income of the family. | 224 | 33% patients had severe Vitamin D deficiency followed by 32% patients with moderate deficiency and 22% with mild deficiency. | 88% | 81.97-  90.79 | Socioeconomic groups in Indian women of reproductive age, development of osteoporosis and pregnancy-related complications. |
| Anu Susan George et al | 2019 | Not given | Kerala, India | Cross-sectional study | Developmental Pediatrics and Child Neurology department of this tertiary care hospital. | Random sampling | Children below 12 y | Below 12 | Neurodevelopmental disorders, demographic data and historical details. | 90 children | (69.2%) girls and (73.4%) | 72.20% | 61.23-  79.62 | Neuro-developmental disabilities, cognitive disability, autistic spectrum disorder. |
| InduLata et al | 2017 | April 2014 and April 2016. | Lucknow, Uttar Pradesh, India | Cross-sectional study | Department of Maternal and Reproductive Health, Sanjay Gandhi Postgraduate Institute of Medical Sciences, Lucknow, Uttar Pradesh, India | Random sampling | Infertile females | 18 - 40 | Age, duration of married life, duration and type of infertility, previous obstetrical history, and education levels were retrieved for all women | 70 | 64.28% | 90% | 52.58-  51.86 | Infertility associated disorders, Dietary intakes, and AMH levels. |
| ManjuDaroach et l | 2017 | Not given | Chandigarh India | Cross-sectional study | Department of Dermatology, Venereology and Leprology, PGIMER, Chandigarh India | Random sampling | Alopecia areata (AA) patients | 30 | Alopecia areata (AA), histopathological examination, | 30 AA patients and 30 healthy controls. | (96.7%) patients and (73.3%) controls | 85% | 73.89-  91.9 | Inflammation, Fibrosis present, Non progressive disease, Progressive disease. |
| John Mechenro et al | 2018 | June 2015 and July 2016 | Tamil Nadu, India. | Cross-sectional study | SRM Medical College Hospital & Research Centre, SRM University, Kancheepuram District, Kattankulathur, Tamil Nadu | Random sampling | Normal village population | Not given | Demographic profile and socioeconomic status Duration of sun exposure, body surface area exposed to sunlight, use of sunscreen while going outdoors, intake of fatty fish, beef, liver and milk. | 424 | 44.80% | 44.80% | 40.13-  49.56 | Gender (females less likely to be sufficient), sunlight exposure (< 60 min pre day less likely to be sufficient), educational qualification (higher education less likely to be sufficient), consumption of fish (non-consumption less likely to be sufficient), and closed toilet system at home (closed toilet less likely to be sufficient). |
| RanadipChowdhury et al | 2017 | January 2010 to February 2012 | Delhi, India | Cross-sectional study | Low-to-middle socioeconomic neighborhoods of Tigri and Dakshinpuri in New Delhi, | Random sampling | Young children. | 6 - 30 months | Neurodevelopment, Communication, Gross motor, Fine motor, Problem-solving and Personal-social | 1000 | 34.50% | Not given | 31.62-  37.5 | None |
| [Ramank K Marwaha et al](https://pubmed.ncbi.nlm.nih.gov/?term=Marwaha+RK&cauthor_id=29470176) | 2018 |  | New Delhi, India | Cross-sectional study | Not found article | Random sampling |  | 6–18 |  | 847 | 94.6% of girls |  | 92.87-  95.93 | Not given |
| Minhaz Husain et al | 2018 |  | Jodhpur, Rajasthan | Cross-sectional study | Department of Pediatrics, Dr. S. N. Medical College, Jodhpur, Rajasthan | Random sampling | Breastfed infants and their mothers. | 1 -30 days | Mothers were evaluated on basis of bone pains, muscle cramp, and signs. Renal function tests, liver function tests, serum Ca, phosphorus, alkaline phosphates, and serum 25(OH)D3 estimation, Ca: creatinine ratio. Infants were evaluated for clinical features of rickets, Serum Ca, phosphorus, alkaline phosphates, and serum 25(OH)D | 200 mother–infant pairs | 47% of mothers and 45% infants | Not given | 41.18-  50.9 | Mothers and infant’s serum 25(OH)D values, Ca levels |
| DiptiSarma et al | 2019 | 2011 to 2013 | Guwahati, Assam | Cross-sectional study | Rural and urban areas of Assam in Northeast India. | Random sampling | School children | 8–14 | Regarding diet, sun exposure, any underlying illness that might interfere with vitamin D synthesis or any intake of drugs or vitamin | 500 students | 85.70% | 8.40% | 82.36-  88.5 | Serum calcium, sun exposure and calcium intake. |
| R Gnanaraj et al | 2020 | Not given | Vellore, South India. | Cross-sectional study | Community in an urban area in Vellore, South India | Random sampling | Healthy infants | 1 | Demographic details including perinatal and neonatal history, vaccination history, breast feeding duration, and history of illnesses, weight, length, mid upper arm circumference | 495 | 22.60% | 22% | 19.13-  26.49 | Breastfeeding for duration more than 6 months, duration of sunlight exposure. |
| A. Gupta et al. | 2018 | Not given | Delhi, India | Cross-sectional study | Department of Medicine, University College of Medical Sciences (University of Delhi) and GTB Hospital, Delhi, India | Random sampling | Type 2 diabetes mellitus patients | Not given | Age and sex, anthropometry, skin-fold thickness, fasting and postprandial plasma glucose, HBA1c, fasting insulin, lipids and vitamin D levels | 213 | 64.3% | 64.3% | 57.66-  70.43 | Diabetic patients |
| Shikha Jain et al. | 2018 | June 2016 to December 2016 | Bhopal, Madhya Pradesh, India. | Cross-sectional study | Tertiary care hospital in Central Kerala | Random sampling | Children | 1 - 5 | Demographic details, detailed clinical history and complete physical examination | 79 | 27.80% | 27.80% | 19.13-  38.53 | Calcium level, PTH levels. |
| SubarnaMitra et al | 2016 | January 2014 to December 2014 | Raipur, India | Cross-sectional study | AIIMS, Raipur, India | Random sampling | Postmenopausal Women | Not given | Clinical and biochemical | 64 | 52% | 52% | 40-  63.78 | Postmenopausal Women, metabolic syndrome. |
| Sanjay GajananGokhale et al | 2017 | November 2014 to December 2016 | Mumbai, India. | Cross-sectional study | Department of Pediatrics, Rajhans Hospital, Mumbai , India. | Random sampling | Women | 19-49 | Pregnancy, ante-natal period, type and details of delivery and weight of baby, a Maternal Weight and Height, BMI, Maternal Hemoglobin | 80 | 60% | 60% | 49.05-  70.04 | Not given |
| RanadipChowdhury et al | 2017 | January 2010 to February 2012 | Delhi, India | Cross-sectional study | Low-to-middle socioeconomic settings of Tigri and Dakshinpuri in New Delhi | Random sampling | Children | 6 - 30 months | Respiratory infections and diarrhea | 960 | 34.50% | 34.50% | 34.48-  31.54 | Age, sex, breastfeeding status, wasting, stunting, underweight, anemia status and season |
| Satheesh Ponnarmeni et al | 2015 |  | Chandigarh, India | Cross-sectional study | Pediatric intensive care unit (PICU), Postgraduate Institute of Medical Education and Research (PGIMER), Chandigarh, India | Random sampling | Critically ill children with sepsis | 1–12 | Demographic data, clinical signs and risk factors for VDD, Pediatric Index of Mortality III (PRISM III) score, and sequential organ failure assessment (SOFA | 613 | 12.1% cases and 8.58% controls | 20.00% | 50.81-  42.12 | PRISM III score, SOFA score, need of mechanical ventilation and catecholamines, shock, MODS, HCAI, hypocalcemia, length of PICU stay, and mortality |
| [RajeshwariDhandai et al](https://pubmed.ncbi.nlm.nih.gov/?term=Dhandai+R&cauthor_id=30003852) | 2018 | September 2015 and February 2016 | New Delhi, India | Cross-sectional study | Tertiary care Pediatric teaching hospital in northern India | Random sampling | Newborn infant | Not given | Physical examination and blood culture, urine culture, chest radiograph and CSF examination. | 60 | 63.35% cases and 50% controls | Not given | 63.33-  50.68 | Correlation between neonatal and maternal vitamin D status |
| [Om PrakashSuthar et al](https://pubmed.ncbi.nlm.nih.gov/?term=Suthar+OP&cauthor_id=30341862) | 2018 | Not given | Jodhpur, Rajasthan. | Cross-sectional study | Regional Geriatric Centre, NPHCE, MDM Hospital attached to Dr. S.N. medical college Jodhpur. | Random sampling | Elderly people | 60 and above | Minor short illness, acute illness, cute coronary events | 100 | Coronary artery disease, 28.3% and healthy subjects, 25.53% | Not given | 27-  19.27 | Arterial stiffness and widening of pulse pressure, Atherosclerosis and cardiovascular morbidity and mortality. |
| AmitAgrawal et al | 2019 | March 2016 to February 2017 | Bhopal, Madhya Pradesh | Cross-sectional study | Department of Pediatrics, Gandhi Medical College, Hamidia Hospital, Bhopal, Madhya Pradesh | Random sampling | Neonates | 72 hours - 21 days | Physical examination and systemic examination findings. | 225 | 81% | Not given | 83.56-  78.16 | Sepsis and sun exposure |
| [Sanjay Prakash et al](https://pubmed.ncbi.nlm.nih.gov/?term=Prakash+S&cauthor_id=28470754) | 2017 | February 2015 and October 2016 | Vadodara, Gujarat | Cross-sectional study | Smt. B. K. Shah Medical Institute and Research Centre, sumandeepvidyapeeth, Piparia, Waghodia, Vadodara, Gujarat | Random sampling | Chronic tension-type headache (CTTH) patients | Not given |  | 157 | 71% | Not given | 71-  61.46 | Musculoskeletal pain, muscle weakness, muscle and bone tenderness score, associated fatigue. |
| Tania Pan et al | 2018 | April–December 2017 | Singur, West Bengal | Cross-sectional study | Village of Singur, West Bengal | Random sampling | Adult females | 40 and above | Sociodemographic characteristics, dietary pattern, their daily sun exposure, tobacco use, and morbidity profile. | 194 | 19.6% | Not given | 14.62-  25.74 | Decreasing age, education up to primary, low SES, the decreasing duration of daily sun exposure, menopause, diabetes, overweight/obesity, and unsatisfactory diet |
| Vivek Dixit et al | 2018 | July 2008 and June 2010 | New Delhi, India | Cross-sectional study | Loknayak Hospital, New Delhi | Random sampling | Postmenopausal women | Not given | Past and present history of illness, previous medications and average duration of sun exposure including surface area of the body exposed daily | 334 | 82.93% | 82.93% | 77.24-  85.51 | PTH levels |
| JhumaSankar et al | 2017 | 6 months. | New Delhi, India | Cross-sectional study | Department of Pediatrics, All India Institute of Medical Sciences, New Delhi, India | Random sampling | Children with septic shock | 17 | diagnosis, demographic variables, illness severity score (pediatric index of mortality-2 [PIM-2]) at admission, duration of sun exposure | 43 | 72% | 72% | 54.9-  81.4 | Septic shock |
| Ziaul Hoda Shaan et al | 2019 | January 2014 to December 2015 | Aligarh, Uttar Pradesh, India | Cross-sectional study | Department of Orthopedics, JNMCH, AMU, Aligarh, Uttar Pradesh, India | Random sampling | Patients with fracture management. | >45 | Low backache, weakness, and generalized body ache without any fracture or comorbidity | 102 | 15.60% | 15.60% | 9.12-  22.86 | PTH levels |
| S Nagarjunakonda et al | 2015 | Month of July 2013 | Guntur, Andhra Pradesh, India. | Cross-sectional study | Department of Neurology, Guntur Medical College, Government General Hospital, Guntur, Andhra Pradesh, India. | Random sampling | Patients with epilepsy | >60 | Clinical and demographic data, orally administered sun exposure, physical activity, dietary vitamin D | 98 | 41% | 41% | 31.61-  50.72 | Varied landscape, sunlight duration, skin color and cultural practices |
| BikrantBihari La et al | 2018 | January 2015 to December 2016 | New Delhi, India | Cross-sectional study | Department of Pediatric Hepatology, Institute of Liver and Biliary Sciences, New Delhi, India | Random sampling | Children with chronic liver diseases | 1 - 18 | Histological and/or radiological evidence | 210 | 70.10% | 70.10% | 63.49-  75.79 | PTH values |
| Sargoor R Veena et al | 2017 | Not given | Mysore, South India | Cross-sectional study | Holdsworth Memorial Hospital, Mysore, South India | Random sampling | Pregnant women and their offsprings | 9 - 14 | Poorer offspring cognitive ability, independent of socio-demographic factors | 663 | 68% | 68% | 64.22-  71.31 | Cognitive performance in their children |
| SuchitraSurve et al | 2018 | November 2015 to March 2017 | Mumbai, Maharashtra, India | Cross-sectional study | Urban slum of the conveniently selected geographical area of Mumbai | Random sampling | Healthy children | 1–5 | [Sociodemographic profile, physical activity profile (sun exposure, time spent outdoor)](https://www.ncbi.nlm.nih.gov/pmc/articles/PMC6085962/#ref17) | 201 | 74.60% | 74.60% | 67.14-  79.24 | Children staying indoors, parathyroid hormone and alkaline phosphatase. |
| Pradeep Kumar Dabla et al | 2016 | April 2013–December 2014 | Delhi, India | Cross-sectional study | Tertiary care Pediatric hospital, GB Pant Hospital, Delhi, India | Random sampling | Osteoarticular tuberculosis patients | <5 or >5 | Economic, educational and health characteristics of the family (e.g., Malnutrition, poor hygiene, infections), age and sex. | 25 | 56% | 56% | 37.07-  73.33 | TB patients |
| Mayank Priyadarshi et al | 2018 | November 2014 and September 2015 | New Delhi, India | Cross-sectional study | Department of Pediatrics, All India Institute of Medical Sciences, New Delhi, India | Random sampling | Infants | 6 months | Estimation of serum 25(OH)D, parathyroid hormone (PTH), calcium, phosphorus, and alkaline phosphatase (ALP) | 70 | 91.30% | 91.30% | 80.77-  95.07 | PTH at birth, hyperparathyroidism |
| RamdossRamu et al | 2018 | November 2012 and February 2014 | New Delhi | Cross-sectional study | PGIMER & Dr. RML Hospital, New Delhi | Random sampling | Patients with hepatic or renal disease | 18 - 64 | Demographic data including age, sex, address and occupation | 40 | 37.50% | 37.50% | 22.13-  50.49 | Serum PTH levels |
| PramilaDharmshaktu et al | 2019 | 2016‐18 | New Delhi | Cross-sectional study | AIIMS, Delhi | Random sampling | Outdoor workers | Not given | Daily dietary intake was assessed a semi‐quantitative food frequency | 605 | 0.00% | 0.00% | 0 | Serum PTH levels |
| Preethi Raja Navaneethan et al | 2015 | Not given | Vellore, Tamil Nadu, India | Cross-sectional study | Department of Obstetrics and Gynecology, Christian Medical College Hospital | Random sampling | Postmenopausal women with pelvic floor disorders |  | Educational status, socioeconomic background, sunlight exposure, obstetric history | 120 | 77.5% | 77.5% | 68.35-  83.34 | Milk intake and calcium supplementation. PFD and Menopausal status. |
| RamamoorthyJayashri et al. | 2020 | 2012 to 2013 | South India | Follow up study | Chennai Urban Rural Epidemiology Study (CURES) | Random | Normal Glucose Tolerant, Prediabetic and Type 2 Diabetic | 20 - 80 | <20ng/ml, Roche e601Cobas immunoassay analyzer | 1500 | 823 | 55% | 52.34-  57.37 | Age, Gender, Height, weight and BMI |
| Sunil Jamwal et al | 2018 | Not given | South Punjab | Cross sectional study | Tertiary Care Centre | Random | Healthy volunteers | 18 - 60 | <20ng/ml, ELISA | 120 | 39 | 32.50% | 24.78-  41.31 | Age, weight, height, body mass index (BMI), and gender |
| Venkataram Shivakumar et al | 2015 | Not given | India | Not given | National Institute of Mental Health and Neurosciences | Random | Schizophrenia patients | 32.14 ± 6.6 | <20ng/ml, immunoassay | 35 | 29 | 83% | 67.32-  91.9 | Age, years of education and total intracranial volume |
| Deba Prasad Dhibar et al | 2016 | March 2012 to May 2012 | Chandigarh | Cross sectional Observational study | Post Graduate Institute of Medical Education and Research (PGIMER) | Consecutively | Patients who underwent coronary angiography | 30 - 70 | <20ng/ml, Electrochemiluminescence immunoassay (ECLIA) | 315 | 263 | 83.50% | 78.99-  87.18 | Age, sex, BMI, and blood pressure |
| Surya Prakash Bhatt et al | 2018 | June 2012 to June 2017 | Delhi | Cross-sectional population-based study | 35 residential locations | Random | Prediabetic women | 20 - 60 | <50nmol/L | 797 | 547 | 68.60% | 65.33-  71.76 | Blood pressure, body mass index (BMI), fasting blood glucose (FBG) and extent of sun exposure |
| Arunkumar Karthikayan et al | 2018 | 2 years | South India | Hospital-based case control study | Tertiary care center | Convenient | Breast cancer patients | 47.1 ± 10.7 | <10ng/ml | 78 | 3 | 3.90% | 1.32-  10.71 | Age, BMI, height, weight, and Hb |
| V Majumdar et al | 2015 | January 2012 to January 2013 | Bangalore | Cross-sectional study | NIMHANS | Random | Patients with ischemic stroke | 47.82±15.28 | <20ng/ml | 239 | 148 | 61.92% | 55.62-  67.84 | Age, gender, BMI, Smoking and alcohol |
| PuneetMisra et al | 2017 | November 2013 to March 2014 | Faridabad district, Haryana | Analytical cross-sectional study | AIIMS | Random | Women in rural community | 20 - 60 | <49.9nmol/L, chemiluminescence method by diasorin LIAISON | 381 | 346 | 90.80% | 87.49-  93.32 | Age, marital status, educational status, occupation, smoking, sun exposure duration, calorie intake, and protein intake, body mass index (BMI), hypertension, and diabetes |
| Nalini Sharma et al | 2019 | 2.5 years | North Eastern India | Prospective study | Antenatal clinic | Consecutive | Pregnant women | 26.71±9.96 | <20ng/ml, Beckman coulter unicell DXI immunoassay system | 177 | 149 | 84.18% | 78.09-  88.82 | No Determinants |
| Poonam Singh et al | 2020 | October, 2015 to September, 2016 | India | Prospective study | Tertiary-care hospital | Not given | Neonates with early onset sepsis | 3 days | <11ng/ml, ECLIA 411 Model with chemiluminescence system attached with ultraviolet detector | 70 | 29 | 41.40% | 30.63-  53.12 | Temperature instability, apnea tachycardia/bradycardia, hypotension, feeding intolerance, abdominal distension, and necrotizing enterocolitis |
| Wasim Ahmad Wani et al. | 2018 | January to December 2016 | Srinagar | Retrospective study | SKIMS hospital | Follow-up | Cystic fibrosis patients | <15 | ≤12ng/ml | 51 | 24 | 47.10% | 34.05-  60.48 | Female sex, number of exacerbations, age at diagnosis, bacterial colonization, and pancreatic enzyme supplementation and that it was negatively correlated to age |
| AbHameedRaina et al | 2016 | 2 years | Srinagar, Kashmir | Prospective case-control study | Sher-i-Kashmir Institute of Medical Sciences (SKIMS) | Not given | Chronic stable angina | Any age group | <20ng/ml, ELISA | 100 | 75 | 75% | 65.7-  82.45 | Age, Gender, BMI, Smoking and Season |
| DeeptiGoswami et al | 2016 | 2011 to 2012 | North India | Case control study | Department of Obstetrics & Gynecology at Maulana Azad Medical College | Not given | Twin Pregnancies | 27.4 ± 4.18 | < 30 nmol/L (12ng/ml) | 50 (maternal), 100(neonatal) | 45 (maternal), 89(neonatal) | 90%(maternal), 89%(neonatal) | 83.37-  93.32 | Age, height, weight and BMI |
| Ambuj Roy et al | 2015 | March 1999 to June 2001 | New Delhi | Case control study conducted | All India Institute of Medical Sciences (AIIMS) | Consecutive | Acute myocardial infarction | 25 - 75 | <30ng/ml, Liaison® 25OH Vitamin D TOTAL (diasorin, USA) | 120 | 118 | 98.30% | 94.12-  99.54 | Age, Tobacco, alcohol and hypertension |
| Jai B Sharma et al | 2019 | Not given | New Delhi | Cross sectional study | Tertiary referral center | Not given | Stress urinary incontinence | 31 - 61 | <20ng/ml, Roche Elecsys 2010 immunoassay analyser | 40 | 30 | 75% | 59.81-  85.81 | Age |
| Gupta Taru et al | 2015 | August 2012 to April 2014 | New Delhi | Case control study | ESIC-PGIMSR | Not given | Preeclampsia | Not given | <15ng/ml | 50 | 45 | 90% | 78.64-  95.65 | BMI, age, mode of delivery, and birth weight |
| BabitaGhai  et al. | 2015 | January 2013 to July 2014 | Chandigarh | Controlled study | Outpatient pain clinic of tertiary care hospital | Single arm clinical trial | Chronic lower back pain | 18 - 75 | ≤20ng/ml, ELECSYS-2010 | 328 | 217 | 66% | 60.88-  71.07 | Age, BMI, Gender and radicular pain |
| Krishnasamy Narayanasamy et al | 2019 | July 2016 to June 2017 | Chennai | Prospective cross-sectional study | OP Unit of the Department of Hepatology at Rajiv Gandhi Government General Hospital | Not given | Patients with chronic liver disease | >18 | 0–19.9 ng/ml | 236 | 87 | 36.90% | 30.96-  43.18 | Age, Gender and alcohol |
| Deepak Oberoi et al | 2019 | June 2016 to April 2017 | India | Case control study | Not given | Not given | Adult CVD patients | 30 - 70 | <30ng/ml, ELISA | 100 | 64 | 64% | 54.24-  72.73 | Smoking and physiological stress |
| Sharma Sheetal et al | 2015 | October 2011 and April 2013 | North India | Prospective cohort study | Loknayak Hospital | Consecutive | Pregnant women | 23.00 ± 5.85 | <32ng/ml, (DLD Diagnostic GMBH | 418 | 391 | 93.50% | 90.76-  95.52 | Maternal education, husband education,SES, serum calcium, serum phosphorous, and season |
| PrakashVipul et al | 2017 | July 2013 to June 2014 | North India | Cross-section single center study | Department of Medicine at tertiary care center | Not given | Patients with sepsis | 18 - 82 | Not given | 88 | 65 | 73.90% | 63.82-  81.9 | Age and Gender |
| Lisa A. Houghton et al | 2019 | September 2014 to March 2015 | Badarpur, New Delhi | Cross-sectional study | Slum area Badarpur, New Delhi | Random sampling |  | Children aged 12–23 months | Not given | 120 | 74% | Not given | 64.78-  80.43 | Anemia, Iron and hemoglobin concentrations |
| K DevarajNaik et al | 2015 | March and April 2013 | Kozhikode, Kerala, India | Cross-sectional study | Tertiary care center, Kozhikode, Kerala | Random sampling | 50 neonates WITH mean birth weight of 2870 g | Neonates, born at term | <20 ng/ml | 50 (22 males) | 47 (94%) | 94% | 83.78-  97.94 | Maternal levels of 25-hydroxy vitamin D levels |
| Kiran Kumar M et al | 2020 | November 2014 to October 2015 | Pondicherry, India | Cross-sectional study | Pediatric Intensive Care Unit, JIPMER Pondicherry, India. | Random sampling | 522 children | Aged 1 month - 12 years | 25(OH)D level < 20 ng/ml | 522 | 29.30% | 30% | 25.21-  32.96 | Exposure to sunlight, climate, skin pigmentation, dietary habits, and underlying medical illness, pregnancy, infant age group, sepsis in children |
| RanadiP et al | 2020 |  |  | Cross-sectional study |  | Random sampling | 791 North Indian children | 6-9 | < 12 ng/ml | 716 | 328 (45.8%) | 45% | 42.19-  49.97 | Early childhood and cognitive development at school age, pregnancy and neurodevelopment |
| NishantRaizada et al | 2020 | January 2008 to February 2017 | North India |  | Tertiary care hospital |  |  |  | <20 ng/ml, LIAISON, diasorin Inc., | 26,339 | 15,593 | 59.20% | 58.61-  59.79 | Year and seasons |
| [Madhava Vijayakumar et al](https://www.cambridge.org/core/search?filters%5BauthorTerms%5D=Madhava%20Vijayakumar&eventCode=SE-AU) | 2019 | 2019 | Kerala, southern | Cross-sectional observational study. | Tertiary government hospital. | Simple Random Sampling | Children | <18 | Sun exposure and Ca and vitamin D intakes | 296 | 11.10% | 10% | 7.76-  14.86 | Lower Sun exposure, Less Vitamin D intakes, Age |
| [Shobhit Garget al](http://www.ijem.in/searchresult.asp?search=&author=Shobhit+Garg&journal=Y&but_search=Search&entries=10&pg=1&s=0) | 2019 | May 2016 to April 2017 | West Bengal, India | Cross-sectional study | Rural Health Unit and Training Center, Singur (RHUTC) | Simple Random Sampling | Adults | >18 | Height, weight, BMI, Physical activity, Daily Sun exposure, Diet | 197 | 15.70% | 15% | 10.45-  20.34 | BMI, Age, lower sun exposure, Vitamin D deficiency |
| Anil Agarwal et al. | 2015 | 2012 to 2014 | Not given | Not given | Tertiary care pediatric center | Not given | Children with osteoarticular tuberculosis (TB) | <5 and 5–12 | <30 nmol/L | 100 | 51 | 51% | 41.35-60.58 | Age, gender and site specific (spinal and non-spinal) |
| A. Ashritha et al | 2019 | September 2016 to June 2018 | Puducherry | Observational case-control study | Tertiary care institute | Not given | Children with Hemophilia | 2 - 18 | <20 ng/ml, | 38 | 36 | 95% | 82.72-98.55 | Age, weight, height and BMI |
| Vijay Prakash Gupta et al | 2019 | Not given | Bareilly, India | Cross-sectional study | Rajshree Medical Research Institute Bareilly | Random sampling | Patients Suffering from Psoriasis | More than 18 | Age, sex, phototype and geographical area | 40 cases and 40 controls | 78% of psoriasis patients and 28% of controls. | Not given | 41.7-63.08 | Not find significant differences |
| Neetu Sharma et al | 2018 | Not given | Himachal Pradesh, India. | Cross-sectional study | Lahaul & Spiti district (mean altitude > 4000 meter), Himachal Pradesh | Random sampling | Adult population | 19-56 |  | 105 | 83.30% | 83.80% | 75.59-89.64 | Geographical location, age. |
| Bhat et al | 2018 |  | Lucknow | Hospitalized based cross-sectional study | Ortho OPD of CIMS |  | Geriatric patients | >60 |  | 200 | 112 | 56% | 49.07-63.7 | Not given |
| BharatiKulkarni etal | 2015 | Not given | Hyderabad, India. | Cross-sectional study | National Institute Nutrition, Hyderabad | Random sampling | Women | 18-20 | Not given | 956 | 33.6% in men and 51.4% women. | High in rural young adults | 81.52-86.08 | Not given |
